# Crystal structure and catalytic activity of tetra­kis­(μ_2_-ethyl 2,6-di-*tert*-butyl-4-methyl­phenyl phos­phato-κ^2^ *O*:*O*′)bis­(ethyl 2,6-di-*tert*-butyl-4-methyl­phenyl phosphato-κ^2^ *O*,*O*′)dilutetium *n*-heptane disolvate

**DOI:** 10.1107/S2056989018004565

**Published:** 2018-03-23

**Authors:** Mikhail E. Minyaev, Alexander N. Tavtorkin, Sof’ya A. Korchagina, Ilya E. Nifant’ev, Andrei V. Churakov

**Affiliations:** a A.V. Topchiev Institute of Petrochemical Synthesis, Russian Academy of Sciences, 29 Leninsky prospect, 119991, Moscow, Russian Federation; b Chemistry Department, M.V. Lomonosov Moscow State University, 1 Leninskie Gory Str., Building 3, Moscow 119991, Russian Federation; c N.S. Kurnakov Institute of General and Inorganic Chemistry, Russian Academy of Sciences, 31 Leninsky Prospect, Moscow 119991, Russian Federation

**Keywords:** crystal structure, lutetium, organophosphate, binuclear complex, coordination compound, acrylo­nitrile polymerization

## Abstract

The title complex, [Lu_2_(C_17_H_28_O_4_P)_6_]·2C_7_H_16_, was formed in the reaction between potassium 2,6-di-*tert*-butyl-4-methyl­phenyl ethyl phosphate, [K(2,6-^*t*^Bu_2_-4-MeC_6_H_2_-O)(EtO)PO_2_], and LuCl_3_(H_2_O)_6_ in water, followed by vacuum drying and recrystallization from heptane. Its crystal structure has triclinic (*P* ) symmetry at 120 K. The lutetium tris­(phosphate) complex has a binuclear [Lu_2_(μ-OPO)_4_] core and the organophosphate ligand exhibits κ^2^ *O*,*O*′ terminal and μ_2_-κ^1^ *O*:κ^1^ *O*′ bridging coordination modes with the Lu^III^ ion being sixfold coordinated. The complex is of inter­est as a precatalyst in the acrylo­nitrile polymerization process and displays good catalytic activity under mild conditions.

## Abstract 2

The title complex {Lu_2_[(2,6-^*t*^Bu_2_-4-MeC_6_H_2_-O)(EtO)PO_2_]_6_}·2(*n*-hepta­ne) contains the binuclear [Lu_2_(μ-OPO)_4_] core and the phosphate ligands display κ^2^ *O*,*O*′ terminal and μ_2_-κ^1^ *O*:κ^1^ *O*′ bridging coordination modes. It demonstrates good catalytic activity in acrylo­nitrile polymerization.

## Chemical context   

Over recent decades, rare-earth complexes bearing organic ligands have been widely used as reagents or catalysts in organic synthesis and especially as catalysts or precatalysts in various polymerization processes (Kobayashi & Anwander, 2001 ▸; Kobayashi *et al.*, 2002 ▸). Rare-earth organophosphates and carboxyl­ates have been successfully applied as catalyst precursors for 1,3-diene polymerization (see Friebe *et al.*, 2006 ▸; Fischbach & Anwander, 2006 ▸; Nifant’ev *et al.*, 2013 ▸, 2014 ▸; Zhang *et al.*, 2010 ▸; Jang *et al.*, 2000 ▸; Kwag, 2002 ▸; Fischbach *et al.*, 2006 ▸; Evans *et al.*, 2001 ▸; Evans & Giarikos, 2004 ▸; Roitershtein *et al.*, 2013 ▸; Wilson 1993 ▸). The use of organic phosphates is not limited to the stereoregular polymerization of conjugated dienes.

Various lanthanide complexes have been applied in the polymerization of heteroatomic polar monomers, including polymerization of methyl methacrylate (Jiang *et al.*, 2000 ▸), *rac*-dilactide (Nifant’ev *et al.*, 2013 ▸) and acrylo­nitrile (Jiang *et al.*, 1997 ▸) under mild conditions. Polymerization methods of obtaining polyacrylo­nitrile or acrylo­nitrile copolymers with other polar monomers, *e.g*. methyl acrylate, may require rather hard conditions (supercritical CO_2_ medium) (Shlyakhtin *et al.*, 2013 ▸; Shlyakhtin *et al.*, 2014*a* ▸,*b* ▸,*c* ▸).

**Figure 1:**
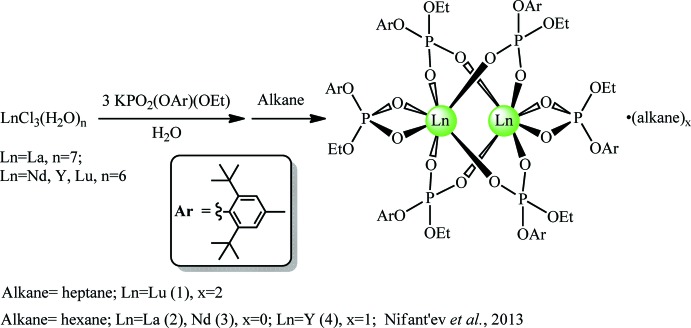
Synthesis of {Ln_2_[(2,6-^*t*^Bu_2_-4-MeC_6_H_2_-O)(EtO)PO_2_]_6_} **1**.

The title complex {Lu_2_[(2,6-^*t*^Bu_2_-4-MeC_6_H_2_-O)(EtO)PO_2_]_6_}·2C_7_H_16_ (**1**), was prepared in the reaction between potassium 2,6-di-*tert*-butyl-4-methyl­phenyl ethyl phosphate, *viz*. [K(2,6-^*t*^Bu_2_-4-MeC_6_H_2_-O)(EtO)PO_2_], and LuCl_3_(H_2_O)_6_ in a 3:1 molar ratio in water followed by vacuum drying and recrystallization from heptane (Fig. 1 ▸), by analogy with the synthesis of {*Ln*_2_[(2,6-^*t*^Bu_2_-4-MeC_6_H_2_-O)(EtO)PO_2_]_6_} [*Ln* = La, CSD refcode TEQCUP (**2**); *Ln* = Nd, TEQDAW (**3**)] and {Y_2_[(2,6-^*t*^Bu_2_-4-MeC_6_H_2_-O)(EtO)PO_2_]_6_}(hexa­ne) [(**4**), TEQDEA] (Fig. 1 ▸), which were earlier obtained by our group (Nifant’ev *et al.*, 2013 ▸). ^1^H and ^31^P{^1^H} NMR studies showed that formation of a binuclear complex occurred upon drying of the aqueous lutetium tris­(phosphate).
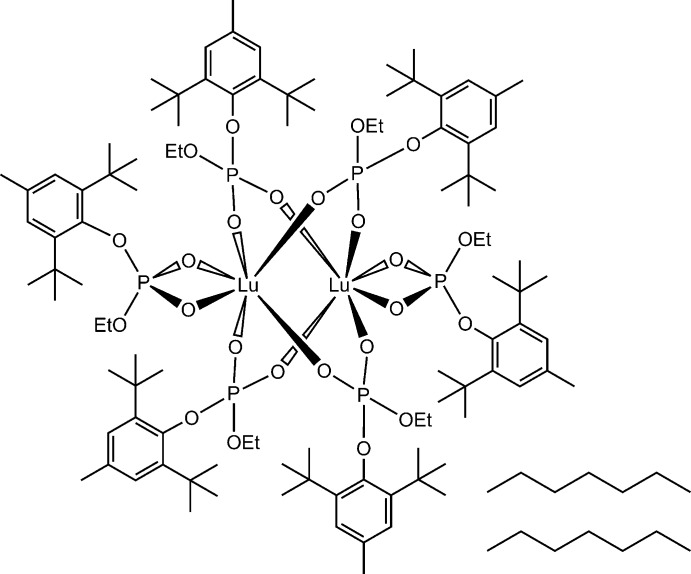


Herein, we report on the crystal structure of the title Lu^III^ tris­(phosphate) complex (**1**), containing the disubstituted organophosphate ligand, and on the catalytic properties of **1** and its Nd analog **3** (see Fig. 1 ▸) in polyacrylo­nitrile synthesis under mild conditions.

## Structural commentary   

**Figure 2:**
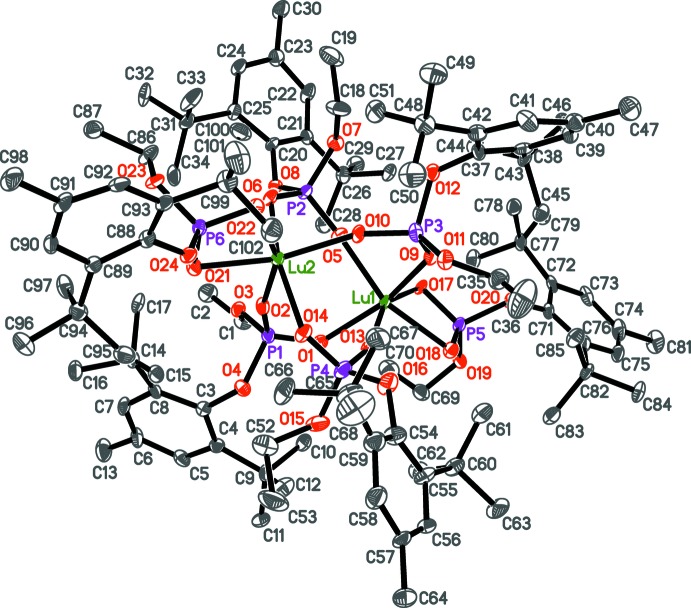
Mol­ecular structure of compound **1** with the atom labelling. Displacement ellipsoids are drawn at the 30% probability level. The solvent mol­ecules and hydrogen atoms have been omitted for clarity.

**Figure 4:**
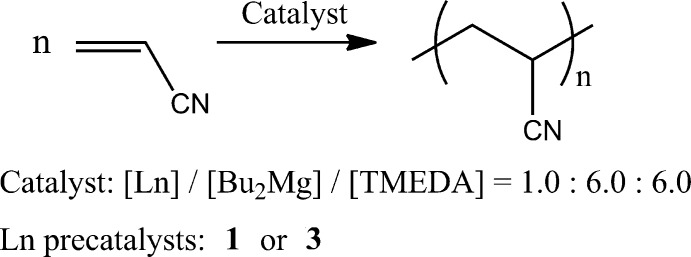
Acrylo­nitrile polymerization reaction.

The title compound, **1**, is a binuclear Lu^III^ tris­(phosphate) complex (Fig. 2 ▸) that crystallized as an *n*-heptane disolvate. The mol­ecular structure of the complex is analogous to those of compounds **2**–**4**. The organophosphate ligand demonstrates κ^2^ *O*,*O*′ terminal and μ_2_-κ^1^ *O*:κ^1^ *O*′ bridging coordination modes (Figs. 2 ▸ and 3 ▸). Most likely, the rather small coordination number for both Lu atoms (CN_Lu_ = 6, a distorted octa­hedron) is induced by steric hindrance of the bulky disubstituted organophosphate ligand. Probably for the same reason, all of the phenyl rings are slightly bent along the C_O_—C_Me_ line with folding angles ranging from 7.9 (6)° (for the OAr substituent at P4) to 8.7 (4)° (OAr at P2) for the bridging phosphates, as well as 6 (1)° (for OAr at P5) and 7.4 (7)° (OAr at P6) for the terminal phosphates. Complex **1** possesses the [Ln_2_(μ-OPO)_4_] core (Fig. 4 ▸) as do complexes **2**–**4**. Ln—O bond distances are presented in Table 1 ▸. As expected, the Lu—O bond distances for the terminal organophosphates are on average 0.07–0.08 Å longer than for the bridging phosphates. The Lu–O–P–O–Lu fragments for all four bridging phosphates are slightly skewed from a symmetrical μ_2_-κ^1^ *O*:κ^1^ *O*′ coordination mode, but not reaching a μ_2_-κ^1^ *O*:κ^2^ *O*,*O*′ semi-bridging coordination mode: *e.g*. Lu1—O1 and Lu2—O2 bond distances (Table 2 ▸) are nearly identical within estimated standard uncertainties, but the Lu1—O2 [3.393 (6) Å] and Lu2—O1 [4.291 (6) Å] distances differ by 0.90 Å. The other bridging ligands demonstrate similar Lu—O distance differences.

The phospho­rous atoms adopt distorted octa­hedral environments. The P—O_Lu_ distances lie in the range of 1.493 (6) Å (P2—O5) to 1.504 (6) Å (P6—O21), whereas the P—O_C_ distances are longer, varying from 1.544 (7) Å (P4—O16) to 1.590 (6) Å (P3—O12). Regardless of aryl steric hindrance, the O_C_—P—O_C_ bond angles [102.2 (3)° for O23—P6—O24 to107.0 (3)° for O11—P3—O12] are generally slightly smaller than the other O—P—O angles [106.1 (4)° for O13—P4—O16 to 114.6 (3)° for O9—P3—O10] with the exceptions of the O_Lu_—P—O_Lu_ angles for the terminal phosphates [105.1 (3)° for O17—P5—O18 and 105.7 (3)° for O21—P6—O22]. However, the O_C_—P—O_C_ bond angle is the smallest within the same PO_4_ fragment for all phosphate ligands. Plausible explanations of these observations have been recently given for rare-earth complexes bearing another bulky disubstituted organophosphate ligand (Minyaev *et al.*, 2017 ▸).

## Catalytic activity   

The catalytic activity of binuclear organophosphate precatalysts was studied in the acrylo­nitrile polymerization reaction. The catalytic system was prepared from either **1** or **3**, *n*-Bu_2_Mg and TMEDA (tetra­methyl­ethylenedi­amine) in a 1:12:12 molar ratio (Fig. 4 ▸, Table 2 ▸), in accordance with the published procedure (Jiang *et al.*, 1997 ▸).

The catalytic system based on **1** (*Ln* = Lu) demonstrated a higher catalytic activity, than the system formed using the precatalyst **3** (*Ln* = Nd). Under equivalent conditions, the polymer yield was twice as high (entries 2 and 3, Table 1 ▸). The higher catalytic activity may be associated with the higher electrophilicity of the lutetium cation due to its smaller ionic radius. Obviously, electrophilic activation significantly accelerates the process, since in the absence of a substantial electrophilic influence (blank experiment, Table 1 ▸, entry 1), polymerization proceeds much more slowly, yielding only 9.6% of the polymer as compared to neodymium (26.0%) and lutetium (48.6%). In the case of **1**, the productivity of the catalytic system is much higher than that for earlier published systems (Jiang *et al.*, 1997 ▸), as well as having polyacrylo­nitrile characteristics which are close to those of commercially available polymers (textile fibres) or of obtained copolymers that may be used in high-quality carbon fibre production (Shlyakhtin *et al.*, 2014*a* ▸).

## Database survey   

Crystal structures of di-substituted organophosphates of rare earths are poorly explored (Minyaev *et al.*, 2017 ▸). Usually, lanthanide organophosphates either do not have a definite composition but possess high catalytic activity or have established crystal structures but exhibit poor catalytic activity because of their coordination polymer structure. The crystal structures of tris­(dialk­yl/di­aryl­phosphate) complexes of rare earths are mainly coordination polymers bearing a dimeth­yl/di­ethyl­phosphate ligand (see the Cambridge Structural Database, V5.38, latest update May 2017; Groom *et al.*, 2016 ▸): {*Ln*[(MeO)_2_PO_2_]_3_}_∞_ (*Ln* = La, CSD refcode: HEBDEX (Zeng *et al.*, 1994 ▸); Nd, LAHREU (Lumetta *et al.*, 2016 ▸); Sm, JEVVOV (Li *et al.*, 1989 ▸); Eu, KIXGON (Li *et al.*, 1991 ▸); {La[(MeO)_2_PO_2_]_3_(H_2_O)}_∞_ (JIGVEA; Liu *et al.*, 1990 ▸); {*Ln*[(EtO)_2_PO_2_]_3_}_∞_ [*Ln* = Nd, BOVREJ and BOVREJ01 (Lebedev *et al.*, 1982 ▸); Ce, JOGJEU (Han *et al.*, 1990 ▸) and KETWUC (Amani *et al.*, 2006 ▸); Pr, JOGJIY (Han *et al.*, 1990 ▸)]. Crystal structures of only three dimeric tris­(phosphate) complexes, **2**–**4** mentioned above, are known (Nifant’ev *et al.*, 2013 ▸): {*Ln*_2_[(2,6-^*t*^Bu_2_-4-MeC_6_H_2_-O)(EtO)PO_2_]_6_} [*Ln* = La (TEQCUP), Nd (TEQDAW)] and {Y_2_[(2,6-^*t*^Bu_2_-4-MeC_6_H_2_-O)(EtO)PO_2_]_6_}(hexa­ne) (TEQDEA). With the exclusion of solvent mol­ecules, their structures are similar to that of **1**.

## Synthesis and crystallization   

### General experimental details   

The synthesis of **1** and polymerization experiments were carried out under a purified argon atmosphere. *n*-Heptane and C_6_D_6_ were distilled over sodium wire. Acrylo­nitrile was distilled over CaH_2_ prior to use. 2,6-Di-*tert*-butyl-4-methyl­phenyl ethyl phospho­ric acid and complex **3** were synthesized according to literature procedures (Nifant’ev *et al.*, 2013 ▸). C/H elemental analysis was performed with a Perkin Elmer 2400 Series II elemental analyser. ^1^H and ^31^P{^1^H} NMR spectra were recorded with a Bruker AVANCE 400 spectrometer at 298 K. Size-exclusion chromatography (SEC) measurements were recorded on an Agilent PL-GPC 220 chromatograph equipped with a PLgel Olexis column (eluent: di­methyl­formamide, 0.01% LiBr, 1 ml min^−1^, 323 K), using universal calibration with a poly(methyl methacrylate) standard. The SEC data were determined by using Kuhn–Mark–Houwink constants for polyacrylo­nitrile.

### Synthesis of complex **1**   

An aqueous solution of KOH (0.19 g, 3.3 mmol in 5 ml) was added in small portions to a stirred suspension of 2,6-di-*tert*-butyl-4-methyl­phenyl ethyl phospho­ric acid (1.01 g, 3.09 mmol) in 10 ml of water until the pH = 7. The resulting solution was filtered. A solution of LuCl_3_(H_2_O)_6_ (0.39 g, 1.0 mmol) in 6 ml of water was added dropwise to the stirred solution of [K(2,6-^*t*^Bu_2_-4-MeC_6_H_2_-O)(EtO)PO_2_]. The formed white suspension was stirred for 3 h. The precipitate was filtered off and dried in air for two days. The yield of Lu[(2,6-^*t*^Bu_2_-4-MeC_6_H_2_-O)(EtO)PO_2_]_3_(H_2_O)_2_ was 1.16 g (0.97 mmol, 97%). ^1^H NMR (400MHz, C_6_D_6_): δ 0.94 (9H, *br s*, OCH_2_CH_3_), 1.73 [54H, *s*, C(CH_3_)_3_], 2.16 (9H, *s*, C_*ipso*_—CH_3_), 4.00 (6H, *br s*, OCH_2_CH_3_), 5.57–6.6 (4H, *br s*, H_2_O), 7.17 (6H, *s*, C_*meta*_—*H*). ^31^P{^1^H} NMR (162MHz, C_6_D_6_): δ −7.5.

Vacuum drying of 1.11 g (0.93 mmol) over P_2_O_5_ resulted in Lu_2_[(2,6-^*t*^Bu_2_-4-MeC_6_H_2_-O)(EtO)PO_2_]_6_. (1.04 g, 0.45 mmol) Calculated for C_102_H_168_Lu_2_O_24_P_6_: C, 52.94%; H, 7.32%. Found: C, 52.82%; H, 7.53%. ^1^H NMR (400MHz, C_6_D_6_): δ 0.67 (12H, *br s*, OCH_2_CH_3_), 1.06 (6H, *br s*, OCH_2_CH_3_), 1.77 [108H, *s*, C(CH_3_)_3_], 2.17 (18H, *s*, C_*ipso*_—CH_3_), 4.05 (12H, *br s*, OCH_2_CH_3_), 7.19 (12H, *br s*, C_*meta*_—H). ^31^P{^1^H} NMR (162 MHz, C_6_D_6_): δ −11.0 (4P, bridging), +0.9 (2P, terminal).

Recrystallization of 0.20 g (0.086 mmol) of Lu_2_[(2,6-^*t*^Bu_2_-4-MeC_6_H_2_-O)(EtO)PO_2_]_6_ from 1 ml of hot heptane led to the formation of crystals of **1**. Some of them were taken for X-ray studies. The remaining crystals were filtered off, washed with cold (273 K) heptane (2 × 0.5 ml) and dried under vacuum, yield 0.08 g. The mother liquor was concentrated to 0.5 ml and cooled to *ca* 253 K overnight. This allowed the isolation of 0.11 g of precipitated crystals. Total yield of **1** was 0.19 g (0.076 mmol, 87%). ^1^H NMR (400MHz, C_6_D_6_): δ 0.64–0.71 (12H, *br m*, OCH_2_CH_3_), 0.90 [12H, *t*, CH_3_(CH_2_)_5_CH_3_], 1.02–1.10 (6H, *br m*, OCH_2_CH_3_), 1.20–1.31 [20H, *m*, CH_3_(CH_2_)_5_CH_3_], 1.76 [108H, *s*, C(CH_3_)_3_], 2.17 (18H, *s*, C_*ipso*_—CH_3_), 3.96–4.15 (12H, *br m*, OCH_2_CH_3_), 7.19 (12H, *br s*, C_*meta*_—H). ^31^P{^1^H} NMR (162 MHz, C_6_D_6_): δ −11.0 (4P, *s*, bridging phosphate), +1.0 (2P, s, terminal phosphate). Calculated for C_116_H_200_Lu_2_O_24_P_6_: C, 55.41%; H, 8.02%. Found: C, 55.70%; H, 8.14%.

### Polymerization experimental details   

**Catalytic system preparation.** The catalyst was obtained by addition of a 1.0 *M* heptane solution of Bu_2_Mg (2.4 ml, 2.4 mmol) to a toluene (7 ml) solution containing 0.2 mmol of either **1** or **3** (which is 0.4 mmol of *Ln*) and TMEDA (0.36 ml, 2.4 mmol). The total volume of the mixture was 10 ml. The mixture was heated at 323 K for 45 min.

**Acrylo­nitrile polymerization**. A glass reactor was charged with toluene (11 ml), acrylo­nitrile (2.19 ml, 33.4 mmol) and the prepared catalytic system (1 ml, containing 0.04 mmol of *Ln*) while stirring at 273 K. The initial acrylo­nitrile/*Ln* molar ratio was 835:1. After 1 h, the reaction was stopped by adding 1 ml of methanol. The polymer was precipitated by 50 ml of acetone. The precipitate was washed with a 1 *M* hydro­chloric acid solution (2 × 10 ml), water (10 ml), acetone (2 × 20 ml), and dried under dynamic vacuum.

## Refinement   

Crystal data, data collection and structure refinement details are summarized in Table 3 ▸. The hydrogen atoms were positioned geometrically (C—H distance = 0.95 Å for aromatic, 0.98 Å for methyl, and 0.99 Å for methyl­ene H atoms) and refined as riding atoms with *U*_iso_(H)= 1.5*U*_eq_(C-meth­yl) and 1.2*U*_eq_(C) for other H atoms. A rotating group model was applied for the methyl groups. Twelve reflections (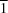 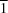 1; 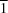 0 1; 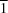 1 0; 0 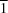 1; 0 0 1; 0 0 2; 0 1 0; 0 1 1; 0 1 2; 1 0 1; 1 1 0; 1 1 1) were affected by the beam stop, and were therefore omitted from the final cycles of refinement. SADI and SIMU *SHELXL* (Sheldrick, 2015*b* ▸) instructions were applied to restrain carbon atoms in the two heptane mol­ecules. One heptane mol­ecule exhibits rather high thermal motions of carbon atoms (C110–C116). The associated disorder could be adequately modelled by using the residual electron density·As a result of these high thermal motions, the final crystallographic model displays rather small inter­molecular H⋯H distances for two neighbouring methyl groups (atoms C110) of inversion-heptane mol­ecules.

## Supplementary Material

Crystal structure: contains datablock(s) I. DOI: 10.1107/S2056989018004565/su5432sup1.cif

Structure factors: contains datablock(s) I. DOI: 10.1107/S2056989018004565/su5432Isup2.hkl

Supporting information file. DOI: 10.1107/S2056989018004565/su5432Isup3.cdx

CCDC reference: 1830858

Additional supporting information:  crystallographic information; 3D view; checkCIF report

## Figures and Tables

**Figure 3:**
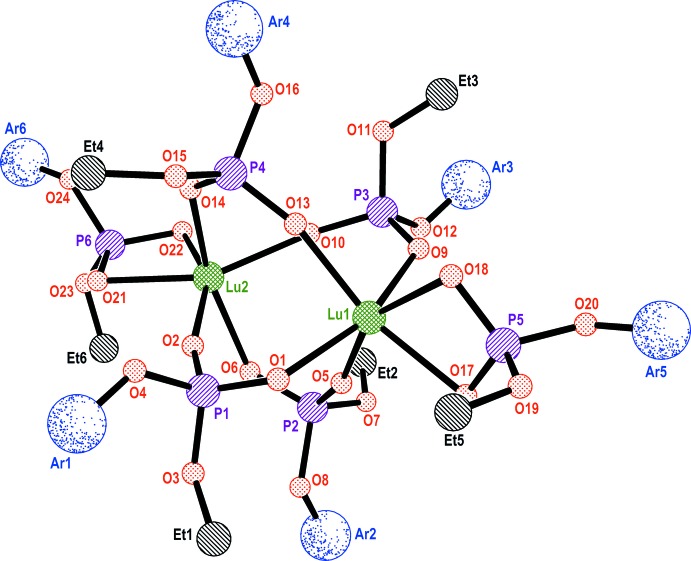
Core atoms in {Lu_2_[(2,6-^*t*^Bu_2_-4-MeC_6_H_2_-O)(EtO)PO_2_]_6_} **1**.

**Table 1:** Selected bond lengths (Å)

Lu1—O1	2.222 (5)	Lu2—O2	2.192 (5)
Lu1—O5	2.196 (5)	Lu2—O6	2.216 (6)
Lu1—O9	2.193 (6)	Lu2—O10	2.178 (5)
Lu1—O13	2.172 (6)	Lu2—O14	2.200 (6)
Lu1—O17	2.280 (5)	Lu2—O21	2.264 (5)
Lu1—O18	2.274 (6)	Lu2—O22	2.276 (5)

**Table 2:** Catalytic activity of **1** or **3** in acrylo­nitrile polymerization *M*_*n*_ and the polydispersity index (PDI) were determined from size-exclusion chromatography (SEC) measurements.

Entry	Precatalyst	Yield, %	*M*_*n* calcd_ × 10^−3^	*M*_*n* found_ × 10^−3^	PDI
1^*a*^	-	9.6	-	12	4.06
2	(1)	48.6	22	33	2.56
3	(3)	26.0	12	13	2.88

Note: (*a*) The blank experiment without a precatalyst.

**Table 3:** Experimental details

Crystal data	
Chemical formula	[Lu_2_(C_17_H_28_O_4_P)_6_]·2C_7_H_16_
*M*_r_	2514.51
Crystal system, space group	Triclinic, *P* 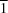
Temperature (K)	120
*a*, *b*, *c* (Å)	14.8828 (15), 19.983 (2), 22.392 (2)
α, β, γ (°)	80.469 (2), 87.417 (2), 74.798 (2)
*V* (Å^3^)	6337.8 (11)
*Z*	2
Radiation type	Mo *K*α
μ (mm^−1^)	1.69
Crystal size (mm)	0.15 × 0.02 × 0.01

Data collection	
Diffractometer	Bruker SMART APEXII
Absorption correction	Multi-scan (*SADABS*; Bruker, 2008 ▸)
*T*_min_, *T*_max_	0.786, 0.983
No. of measured, independent and observed [*I* > 2σ(*I*)] reflections	46151, 24296, 13178
*R*_int_	0.099
(sin θ/λ)_max_ (Å^−1^)	0.617

Refinement	
*R*[*F* ^2^ > 2σ(*F* ^2^)], *wR*(*F* ^2^), *S*	0.063, 0.147, 0.96
No. of reflections	24296
No. of parameters	1386
No. of restraints	74
H-atom treatment	H-atom parameters constrained
Δρ_max_, Δρ_min_ (e Å^−3^)	1.42, −1.50

Computer programs: *APEX2* and *SAINT* (Bruker, 2008 ▸), *SHELXT* (Sheldrick, 2015*a* ▸), *SHELXL2017/1* (Sheldrick, 2015*b* ▸), *SHELXTL* (Sheldrick, 2008 ▸) and *publCIF* (Westrip, 2010 ▸).

## supplementary crystallographic information

### Crystal data

**Table:**

[Lu_2_(C_17_H_28_O_4_P)_6_]·2C_7_H_16_	*Z* = 2
*M**_r_* = 2514.51	*F*(000) = 2640
Triclinic, *P*1	*D*_x_ = 1.318 Mg m^−^^3^
*a* = 14.8828 (15) Å	Mo *K*α radiation, λ = 0.71073 Å
*b* = 19.983 (2) Å	Cell parameters from 3700 reflections
*c* = 22.392 (2) Å	θ = 2.2–20.0°
α = 80.469 (2)°	µ = 1.69 mm^−^^1^
β = 87.417 (2)°	*T* = 120 K
γ = 74.798 (2)°	Needle, colourless
*V* = 6337.8 (11) Å^3^	0.15 × 0.02 × 0.01 mm

### Data collection

**Table:**

Bruker SMART APEXII diffractometer	24296 independent reflections
Radiation source: fine-focus sealed tube	13178 reflections with *I* > 2σ(*I*)
Graphite monochromator	*R*_int_ = 0.099
ω scans	θ_max_ = 26.0°, θ_min_ = 1.4°
Absorption correction: multi-scan (SADABS; Bruker, 2008)	*h* = −18→16
*T*_min_ = 0.786, *T*_max_ = 0.983	*k* = −24→24
46151 measured reflections	*l* = −27→26

### Refinement

**Table:**

Refinement on *F*^2^	Secondary atom site location: difference Fourier map
Least-squares matrix: full	Hydrogen site location: inferred from neighbouring sites
*R*[*F*^2^ > 2σ(*F*^2^)] = 0.063	H-atom parameters constrained
*wR*(*F*^2^) = 0.147	*w* = 1/[σ^2^(*F*_o_^2^) + (0.0535*P*)^2^] where *P* = (*F*_o_^2^ + 2*F*_c_^2^)/3
*S* = 0.96	(Δ/σ)_max_ = 0.001
24296 reflections	Δρ_max_ = 1.42 e Å^−^^3^
1386 parameters	Δρ_min_ = −1.50 e Å^−^^3^
74 restraints	Extinction correction: (SHELXL-2017/1; Sheldrick, 2015b), Fc^*^=kFc[1+0.001xFc^2^λ^3^/sin(2θ)]^-1/4^
Primary atom site location: structure-invariant direct methods	Extinction coefficient: 0.00024 (6)

### Special details

**Table:**

Geometry. All esds (except the esd in the dihedral angle between two l.s. planes) are estimated using the full covariance matrix. The cell esds are taken into account individually in the estimation of esds in distances, angles and torsion angles; correlations between esds in cell parameters are only used when they are defined by crystal symmetry. An approximate (isotropic) treatment of cell esds is used for estimating esds involving l.s. planes.
Refinement. Refinement of F^2^ against ALL reflections. The weighted R-factor wR and goodness of fit S are based on F^2^, conventional R-factors R are based on F, with F set to zero for negative F^2^. The threshold expression of F^2^ > 2sigma(F^2^) is used only for calculating R-factors(gt) etc. and is not relevant to the choice of reflections for refinement. R-factors based on F^2^ are statistically about twice as large as those based on F, and R- factors based on ALL data will be even larger.

### Fractional atomic coordinates and isotropic or equivalent isotropic displacement parameters (Å^2^)

**Table:**

	*x*	*y*	*z*	*U*_iso_*/*U*_eq_	
Lu1	0.84389 (3)	0.20120 (2)	0.15532 (2)	0.02069 (11)	
Lu2	0.65941 (3)	0.20699 (2)	0.29484 (2)	0.02032 (11)	
P1	0.83582 (16)	0.05847 (12)	0.25062 (10)	0.0258 (5)	
P2	0.60763 (16)	0.17844 (12)	0.16119 (10)	0.0244 (5)	
P3	0.69659 (18)	0.34982 (12)	0.18281 (11)	0.0305 (6)	
P4	0.87138 (18)	0.22576 (14)	0.30977 (11)	0.0355 (6)	
P5	0.96535 (16)	0.20706 (12)	0.05347 (10)	0.0260 (6)	
P6	0.53335 (16)	0.21743 (12)	0.39372 (10)	0.0242 (5)	
O1	0.8890 (4)	0.0901 (3)	0.2003 (3)	0.0280 (14)	
O2	0.7598 (4)	0.1105 (3)	0.2769 (2)	0.0290 (14)	
O3	0.7924 (4)	0.0041 (3)	0.2286 (3)	0.0275 (14)	
O4	0.9042 (4)	0.0166 (3)	0.3045 (2)	0.0297 (15)	
O5	0.7058 (4)	0.1826 (3)	0.1496 (3)	0.0281 (14)	
O6	0.5784 (4)	0.1802 (3)	0.2258 (2)	0.0258 (14)	
O7	0.5398 (4)	0.2377 (3)	0.1184 (3)	0.0296 (15)	
O8	0.5991 (4)	0.1085 (3)	0.1402 (2)	0.0253 (14)	
O9	0.7678 (4)	0.3125 (3)	0.1416 (3)	0.0309 (15)	
O10	0.6615 (4)	0.3022 (3)	0.2320 (3)	0.0314 (15)	
O11	0.7352 (4)	0.4019 (3)	0.2134 (3)	0.0351 (16)	
O12	0.6080 (4)	0.3956 (3)	0.1443 (3)	0.0304 (15)	
O13	0.8756 (4)	0.2209 (3)	0.2435 (3)	0.0352 (16)	
O14	0.7780 (4)	0.2263 (3)	0.3382 (2)	0.0291 (15)	
O15	0.9508 (4)	0.1649 (4)	0.3435 (3)	0.051 (2)	
O16	0.8977 (5)	0.2943 (4)	0.3153 (3)	0.0443 (18)	
O17	0.8781 (4)	0.1818 (3)	0.0584 (2)	0.0253 (14)	
O18	0.9759 (4)	0.2266 (3)	0.1142 (2)	0.0289 (14)	
O19	1.0542 (4)	0.1521 (3)	0.0350 (3)	0.0296 (15)	
O20	0.9555 (4)	0.2702 (3)	0.0007 (2)	0.0262 (14)	
O21	0.6107 (4)	0.1548 (3)	0.3834 (2)	0.0263 (14)	
O22	0.5349 (4)	0.2728 (3)	0.3401 (2)	0.0264 (14)	
O23	0.4355 (4)	0.1997 (3)	0.4048 (2)	0.0279 (14)	
O24	0.5478 (4)	0.2435 (3)	0.4540 (2)	0.0267 (14)	
C1	0.8424 (6)	−0.0465 (4)	0.1899 (4)	0.032 (2)	
H1A	0.902150	−0.074524	0.208944	0.039*	
H1B	0.855837	−0.021398	0.149965	0.039*	
C2	0.7824 (7)	−0.0932 (5)	0.1824 (4)	0.043 (3)	
H2A	0.810135	−0.123105	0.152316	0.065*	
H2B	0.720450	−0.064646	0.168707	0.065*	
H2C	0.776799	−0.122746	0.221241	0.065*	
C3	0.9307 (6)	−0.0581 (4)	0.3247 (4)	0.024 (2)	
C4	1.0203 (6)	−0.0934 (5)	0.3100 (4)	0.028 (2)	
C5	1.0389 (7)	−0.1661 (5)	0.3210 (4)	0.035 (2)	
H5	1.099060	−0.192777	0.311524	0.042*	
C6	0.9741 (7)	−0.2015 (5)	0.3452 (4)	0.038 (2)	
C7	0.8913 (6)	−0.1625 (4)	0.3646 (4)	0.032 (2)	
H7	0.848524	−0.187064	0.384066	0.038*	
C8	0.8654 (6)	−0.0896 (4)	0.3579 (4)	0.028 (2)	
C9	1.0990 (6)	−0.0584 (5)	0.2866 (4)	0.034 (2)	
C10	1.0878 (7)	−0.0328 (5)	0.2178 (4)	0.047 (3)	
H10A	1.143698	−0.018827	0.201712	0.071*	
H10B	1.033379	0.007566	0.210217	0.071*	
H10C	1.079365	−0.070799	0.197884	0.071*	
C11	1.1942 (6)	−0.1116 (5)	0.2955 (5)	0.045 (3)	
H11A	1.242786	−0.089215	0.278176	0.067*	
H11B	1.195034	−0.151610	0.275172	0.067*	
H11C	1.205795	−0.128124	0.338855	0.067*	
C12	1.1023 (7)	0.0010 (5)	0.3201 (5)	0.056 (3)	
H12A	1.160246	0.015003	0.310069	0.084*	
H12B	1.099845	−0.014864	0.363865	0.084*	
H12C	1.049010	0.041219	0.308174	0.084*	
C13	0.9962 (8)	−0.2806 (5)	0.3515 (5)	0.061 (3)	
H13A	0.993436	−0.300740	0.394279	0.092*	
H13B	1.058879	−0.298629	0.335666	0.092*	
H13C	0.950730	−0.293716	0.328520	0.092*	
C14	0.7770 (6)	−0.0530 (5)	0.3906 (4)	0.033 (2)	
C15	0.7909 (6)	0.0093 (5)	0.4155 (4)	0.033 (2)	
H15A	0.804626	0.043732	0.382225	0.049*	
H15B	0.842958	−0.006307	0.444150	0.049*	
H15C	0.734083	0.031052	0.436401	0.049*	
C16	0.7563 (7)	−0.1035 (5)	0.4461 (4)	0.041 (3)	
H16A	0.710091	−0.077104	0.471914	0.062*	
H16B	0.813727	−0.126083	0.469068	0.062*	
H16C	0.731758	−0.139504	0.432596	0.062*	
C17	0.6904 (6)	−0.0308 (5)	0.3505 (4)	0.034 (2)	
H17A	0.696603	0.007120	0.317921	0.051*	
H17B	0.635210	−0.014157	0.374872	0.051*	
H17C	0.683854	−0.070993	0.332845	0.051*	
C18	0.4678 (7)	0.2937 (6)	0.1360 (5)	0.050 (3)	
H18A	0.487632	0.337831	0.126817	0.061*	
H18B	0.458126	0.283969	0.180336	0.061*	
C19	0.3807 (8)	0.3029 (6)	0.1056 (5)	0.066 (4)	
H19A	0.333099	0.340777	0.120070	0.098*	
H19B	0.361003	0.259138	0.114255	0.098*	
H19C	0.389056	0.314910	0.061830	0.098*	
C20	0.5338 (6)	0.1031 (4)	0.0988 (4)	0.023 (2)	
C21	0.5565 (6)	0.1102 (4)	0.0373 (4)	0.025 (2)	
C22	0.4849 (7)	0.1181 (5)	−0.0027 (4)	0.036 (2)	
H22	0.497648	0.125309	−0.044861	0.044*	
C23	0.3967 (7)	0.1159 (5)	0.0155 (4)	0.037 (2)	
C24	0.3803 (6)	0.1003 (5)	0.0772 (4)	0.034 (2)	
H24	0.320038	0.096609	0.090487	0.040*	
C25	0.4495 (6)	0.0899 (5)	0.1204 (4)	0.027 (2)	
C26	0.6565 (6)	0.1098 (5)	0.0106 (4)	0.031 (2)	
C27	0.6684 (6)	0.1848 (4)	−0.0007 (4)	0.033 (2)	
H27A	0.732352	0.183804	−0.013995	0.049*	
H27B	0.655796	0.204856	0.036837	0.049*	
H27C	0.624791	0.213658	−0.032138	0.049*	
C28	0.7343 (6)	0.0615 (5)	0.0511 (4)	0.037 (2)	
H28A	0.793430	0.055486	0.028955	0.056*	
H28B	0.720884	0.015693	0.063249	0.056*	
H28C	0.738618	0.082261	0.087233	0.056*	
C29	0.6683 (7)	0.0821 (5)	−0.0500 (4)	0.042 (3)	
H29A	0.732315	0.078142	−0.064615	0.063*	
H29B	0.624846	0.114756	−0.079725	0.063*	
H29C	0.655441	0.035864	−0.044414	0.063*	
C30	0.3187 (7)	0.1296 (6)	−0.0298 (4)	0.051 (3)	
H30A	0.332916	0.093061	−0.055565	0.076*	
H30B	0.312673	0.175646	−0.054935	0.076*	
H30C	0.260185	0.129073	−0.008084	0.076*	
C31	0.4268 (6)	0.0666 (5)	0.1879 (4)	0.034 (2)	
C32	0.3607 (7)	0.0185 (5)	0.1903 (4)	0.047 (3)	
H32A	0.353718	−0.002824	0.232348	0.071*	
H32B	0.386545	−0.018496	0.165873	0.071*	
H32C	0.299727	0.046281	0.174210	0.071*	
C33	0.3757 (7)	0.1302 (6)	0.2167 (5)	0.055 (3)	
H33A	0.416934	0.161084	0.217565	0.082*	
H33B	0.357268	0.114478	0.258106	0.082*	
H33C	0.320107	0.155996	0.192834	0.082*	
C34	0.5124 (7)	0.0250 (6)	0.2244 (4)	0.053 (3)	
H34A	0.547131	0.057152	0.234258	0.079*	
H34B	0.552009	−0.008470	0.200700	0.079*	
H34C	0.493308	−0.000595	0.261959	0.079*	
C35	0.7983 (7)	0.4396 (5)	0.1820 (5)	0.046 (3)	
H35A	0.771663	0.464331	0.142210	0.055*	
H35B	0.858148	0.405900	0.174839	0.055*	
C36	0.8154 (10)	0.4919 (7)	0.2183 (7)	0.108 (5)	
H36A	0.849780	0.522140	0.193511	0.162*	
H36B	0.851954	0.466926	0.254335	0.162*	
H36C	0.755685	0.520675	0.230621	0.162*	
C37	0.5900 (6)	0.4687 (4)	0.1233 (4)	0.026 (2)	
C38	0.6229 (6)	0.4907 (4)	0.0646 (4)	0.026 (2)	
C39	0.6142 (6)	0.5629 (5)	0.0507 (4)	0.032 (2)	
H39	0.636160	0.580523	0.012373	0.038*	
C40	0.5758 (7)	0.6105 (5)	0.0893 (4)	0.036 (2)	
C41	0.5370 (7)	0.5857 (4)	0.1428 (4)	0.037 (3)	
H41	0.507827	0.618175	0.168752	0.045*	
C42	0.5386 (6)	0.5156 (5)	0.1604 (4)	0.033 (2)	
C43	0.6628 (6)	0.4448 (4)	0.0168 (4)	0.029 (2)	
C44	0.6274 (6)	0.3784 (4)	0.0206 (4)	0.032 (2)	
H44A	0.559408	0.391002	0.024423	0.049*	
H44B	0.654808	0.344498	0.056026	0.049*	
H44C	0.645460	0.357477	−0.016148	0.049*	
C45	0.7703 (6)	0.4244 (4)	0.0211 (4)	0.031 (2)	
H45A	0.792304	0.467134	0.016722	0.046*	
H45B	0.796952	0.397658	−0.011322	0.046*	
H45C	0.789796	0.395668	0.060444	0.046*	
C46	0.6368 (7)	0.4860 (5)	−0.0469 (4)	0.038 (2)	
H46A	0.568858	0.500883	−0.050993	0.057*	
H46B	0.663221	0.455937	−0.077165	0.057*	
H46C	0.661687	0.527412	−0.053290	0.057*	
C47	0.5721 (8)	0.6866 (5)	0.0715 (5)	0.054 (3)	
H47A	0.537811	0.712931	0.102315	0.082*	
H47B	0.540582	0.704395	0.032395	0.082*	
H47C	0.635607	0.692468	0.068179	0.082*	
C48	0.4841 (7)	0.4956 (5)	0.2188 (4)	0.038 (2)	
C49	0.3979 (7)	0.5554 (5)	0.2232 (5)	0.052 (3)	
H49A	0.357244	0.540481	0.255453	0.079*	
H49B	0.364402	0.568084	0.184563	0.079*	
H49C	0.416344	0.596175	0.232392	0.079*	
C50	0.5427 (8)	0.4850 (6)	0.2746 (5)	0.064 (4)	
H50A	0.504436	0.478653	0.310869	0.097*	
H50B	0.566582	0.526314	0.274571	0.097*	
H50C	0.594926	0.443306	0.274696	0.097*	
C51	0.4496 (7)	0.4296 (5)	0.2173 (5)	0.050 (3)	
H51A	0.407618	0.423540	0.251579	0.076*	
H51B	0.502957	0.388318	0.219950	0.076*	
H51C	0.416150	0.435056	0.179314	0.076*	
C52	0.9445 (8)	0.1236 (6)	0.4063 (5)	0.061 (3)	
H52A	0.885155	0.144586	0.425937	0.073*	
H52B	0.946396	0.074392	0.403294	0.073*	
C53	1.0243 (9)	0.1257 (7)	0.4425 (6)	0.086 (5)	
H53A	1.020801	0.100062	0.483482	0.129*	
H53B	1.022321	0.174656	0.444801	0.129*	
H53C	1.082680	0.103847	0.423296	0.129*	
C54	0.9674 (7)	0.3003 (5)	0.3551 (4)	0.041 (3)	
C55	1.0617 (6)	0.2896 (5)	0.3354 (5)	0.040 (3)	
C56	1.1223 (6)	0.2930 (5)	0.3796 (4)	0.034 (2)	
H56	1.185904	0.287310	0.368480	0.041*	
C57	1.0994 (7)	0.3034 (5)	0.4364 (4)	0.035 (2)	
C58	1.0067 (8)	0.3193 (6)	0.4520 (5)	0.053 (3)	
H58	0.989925	0.328096	0.491923	0.063*	
C59	0.9355 (7)	0.3230 (6)	0.4113 (5)	0.046 (3)	
C60	1.0964 (6)	0.2790 (5)	0.2713 (4)	0.035 (2)	
C61	1.0278 (7)	0.3276 (5)	0.2230 (4)	0.046 (3)	
H61A	0.968021	0.315277	0.227150	0.069*	
H61B	1.053415	0.321827	0.182506	0.069*	
H61C	1.018442	0.376549	0.228641	0.069*	
C62	1.1134 (7)	0.2024 (5)	0.2612 (4)	0.045 (3)	
H62A	1.053582	0.190727	0.260167	0.067*	
H62B	1.151348	0.171146	0.294417	0.067*	
H62C	1.146135	0.196530	0.222730	0.067*	
C63	1.1899 (7)	0.2986 (6)	0.2588 (5)	0.054 (3)	
H63A	1.238854	0.264163	0.283629	0.081*	
H63B	1.184220	0.345425	0.268929	0.081*	
H63C	1.206120	0.298759	0.215854	0.081*	
C64	1.1695 (8)	0.3047 (6)	0.4815 (5)	0.053 (3)	
H64A	1.230814	0.276578	0.470931	0.079*	
H64B	1.151592	0.285123	0.521930	0.079*	
H64C	1.172050	0.353266	0.481160	0.079*	
C65	0.8319 (7)	0.3478 (6)	0.4287 (5)	0.050 (3)	
C66	0.7984 (7)	0.2830 (6)	0.4635 (5)	0.054 (3)	
H66A	0.797884	0.250242	0.435528	0.081*	
H66B	0.735543	0.299569	0.479493	0.081*	
H66C	0.841129	0.259108	0.497004	0.081*	
C67	0.7704 (7)	0.3870 (6)	0.3761 (5)	0.060 (3)	
H67A	0.791714	0.428285	0.357923	0.090*	
H67B	0.706110	0.402108	0.390223	0.090*	
H67C	0.773408	0.356244	0.345847	0.090*	
C68	0.8228 (10)	0.3982 (7)	0.4766 (7)	0.095 (5)	
H68A	0.850547	0.436854	0.460300	0.143*	
H68B	0.855279	0.372072	0.513774	0.143*	
H68C	0.756836	0.417247	0.485580	0.143*	
C69	1.0917 (7)	0.0871 (5)	0.0743 (4)	0.039 (3)	
H69A	1.075826	0.092699	0.116903	0.047*	
H69B	1.160376	0.073105	0.070444	0.047*	
C70	1.0517 (7)	0.0327 (5)	0.0570 (5)	0.051 (3)	
H70A	1.074935	−0.011665	0.084490	0.077*	
H70B	1.070061	0.025966	0.015390	0.077*	
H70C	0.983671	0.047579	0.059728	0.077*	
C71	1.0134 (6)	0.2818 (4)	−0.0495 (4)	0.026 (2)	
C72	0.9917 (6)	0.2653 (4)	−0.1050 (4)	0.031 (2)	
C73	1.0527 (7)	0.2730 (4)	−0.1528 (4)	0.037 (2)	
H73	1.042073	0.259462	−0.190048	0.044*	
C74	1.1280 (8)	0.2996 (5)	−0.1482 (5)	0.044 (3)	
C75	1.1420 (6)	0.3204 (5)	−0.0943 (5)	0.038 (3)	
H75	1.193577	0.339199	−0.091062	0.046*	
C76	1.0829 (6)	0.3147 (5)	−0.0443 (4)	0.033 (2)	
C77	0.9036 (6)	0.2416 (5)	−0.1166 (4)	0.033 (2)	
C78	0.8147 (6)	0.2877 (5)	−0.0907 (4)	0.035 (2)	
H78A	0.810544	0.337353	−0.105563	0.052*	
H78B	0.817514	0.279041	−0.046407	0.052*	
H78C	0.759700	0.275738	−0.103920	0.052*	
C79	0.8871 (8)	0.2479 (6)	−0.1846 (4)	0.048 (3)	
H79A	0.889644	0.294701	−0.204979	0.072*	
H79B	0.825743	0.240804	−0.191010	0.072*	
H79C	0.935332	0.212171	−0.201310	0.072*	
C80	0.9163 (7)	0.1633 (4)	−0.0895 (4)	0.038 (2)	
H80A	0.864104	0.147275	−0.101772	0.057*	
H80B	0.918179	0.157686	−0.045227	0.057*	
H80C	0.974741	0.135280	−0.104367	0.057*	
C81	1.1943 (7)	0.3064 (6)	−0.2018 (4)	0.053 (3)	
H81A	1.219160	0.260382	−0.214485	0.080*	
H81B	1.245698	0.323397	−0.189719	0.080*	
H81C	1.160443	0.339681	−0.235588	0.080*	
C82	1.1036 (6)	0.3432 (5)	0.0127 (4)	0.035 (2)	
C83	1.1596 (6)	0.2834 (5)	0.0592 (4)	0.039 (2)	
H83A	1.168604	0.301855	0.095751	0.058*	
H83B	1.220329	0.262968	0.041941	0.058*	
H83C	1.125579	0.247168	0.069567	0.058*	
C84	1.1600 (7)	0.3967 (5)	−0.0031 (5)	0.047 (3)	
H84A	1.164020	0.418618	0.032434	0.071*	
H84B	1.129685	0.432915	−0.036311	0.071*	
H84C	1.222759	0.373357	−0.015584	0.071*	
C85	1.0129 (6)	0.3813 (5)	0.0410 (4)	0.038 (2)	
H85A	1.027348	0.405876	0.072325	0.058*	
H85B	0.979013	0.347044	0.059282	0.058*	
H85C	0.974315	0.415423	0.009652	0.058*	
C86	0.4057 (6)	0.1639 (6)	0.3622 (4)	0.041 (3)	
H86A	0.459056	0.127670	0.349601	0.049*	
H86B	0.379496	0.197594	0.325716	0.049*	
C87	0.3325 (6)	0.1303 (5)	0.3929 (4)	0.039 (3)	
H87A	0.308107	0.107460	0.364105	0.059*	
H87B	0.281652	0.166331	0.407045	0.059*	
H87C	0.360193	0.095128	0.427517	0.059*	
C88	0.4850 (6)	0.2517 (5)	0.5047 (4)	0.025 (2)	
C89	0.4951 (6)	0.1942 (5)	0.5514 (4)	0.026 (2)	
C90	0.4280 (6)	0.2027 (4)	0.5968 (4)	0.028 (2)	
H90	0.427824	0.164313	0.628120	0.034*	
C91	0.3613 (6)	0.2660 (6)	0.5976 (4)	0.037 (3)	
C92	0.3630 (6)	0.3212 (5)	0.5547 (4)	0.031 (2)	
H92	0.318800	0.364650	0.557000	0.037*	
C93	0.4267 (6)	0.3176 (4)	0.5070 (4)	0.026 (2)	
C94	0.5730 (6)	0.1247 (5)	0.5560 (4)	0.032 (2)	
C95	0.6675 (6)	0.1372 (5)	0.5382 (4)	0.040 (3)	
H95A	0.669057	0.152744	0.494361	0.059*	
H95B	0.716039	0.093427	0.549168	0.059*	
H95C	0.678393	0.173394	0.559446	0.059*	
C96	0.5821 (7)	0.0863 (5)	0.6233 (4)	0.047 (3)	
H96A	0.633948	0.043943	0.626607	0.070*	
H96B	0.524198	0.073223	0.635802	0.070*	
H96C	0.593696	0.117804	0.649553	0.070*	
C97	0.5482 (7)	0.0748 (4)	0.5184 (4)	0.040 (3)	
H97A	0.549989	0.093933	0.475271	0.060*	
H97B	0.485492	0.069734	0.529241	0.060*	
H97C	0.593157	0.028755	0.526575	0.060*	
C98	0.2884 (7)	0.2728 (6)	0.6488 (4)	0.047 (3)	
H98A	0.225884	0.290638	0.631277	0.071*	
H98B	0.299236	0.305517	0.674150	0.071*	
H98C	0.293806	0.226691	0.673608	0.071*	
C99	0.4251 (7)	0.3849 (5)	0.4606 (4)	0.039 (2)	
C100	0.3710 (7)	0.3885 (5)	0.4039 (4)	0.041 (3)	
H10D	0.398942	0.347213	0.384647	0.062*	
H10E	0.372820	0.431191	0.375726	0.062*	
H10F	0.306182	0.389310	0.414700	0.062*	
C101	0.3761 (9)	0.4520 (5)	0.4879 (5)	0.064 (4)	
H10G	0.380932	0.493790	0.459351	0.096*	
H10H	0.406164	0.451277	0.526124	0.096*	
H10I	0.310306	0.453334	0.495327	0.096*	
C102	0.5242 (7)	0.3926 (5)	0.4446 (4)	0.046 (3)	
H10J	0.549989	0.365244	0.412458	0.069*	
H10K	0.563903	0.375240	0.480585	0.069*	
H10L	0.521659	0.442193	0.430683	0.069*	
C103	0.7304 (9)	0.3673 (9)	0.6413 (6)	0.107 (6)	
H13D	0.785922	0.368061	0.616159	0.161*	
H13E	0.748763	0.337029	0.680207	0.161*	
H13F	0.699317	0.415024	0.648173	0.161*	
C104	0.6660 (9)	0.3395 (7)	0.6099 (6)	0.087 (5)	
H14D	0.701228	0.294554	0.597675	0.104*	
H14E	0.643824	0.372888	0.572491	0.104*	
C105	0.5830 (9)	0.3274 (6)	0.6458 (5)	0.075 (4)	
H15D	0.548536	0.372367	0.658413	0.089*	
H15E	0.541647	0.314647	0.618698	0.089*	
C106	0.6007 (7)	0.2728 (5)	0.7006 (5)	0.058 (3)	
H16D	0.637640	0.287810	0.729049	0.070*	
H16E	0.640111	0.229031	0.688414	0.070*	
C107	0.5190 (7)	0.2555 (5)	0.7344 (4)	0.056 (3)	
H17D	0.484164	0.297560	0.751437	0.068*	
H17E	0.477495	0.246835	0.704828	0.068*	
C108	0.5373 (8)	0.1946 (6)	0.7845 (5)	0.072 (4)	
H18D	0.578036	0.153644	0.768648	0.086*	
H18E	0.572394	0.205603	0.816422	0.086*	
C109	0.4545 (8)	0.1737 (7)	0.8130 (6)	0.078 (4)	
H19D	0.474794	0.133648	0.845444	0.118*	
H19E	0.420287	0.160385	0.782425	0.118*	
H19F	0.413870	0.213221	0.829851	0.118*	
C110	0.0023 (18)	0.5193 (13)	0.4373 (9)	0.252 (8)	
H10M	−0.007577	0.478107	0.464508	0.378*	
H10N	−0.041076	0.561851	0.447776	0.378*	
H10O	0.066441	0.522143	0.441453	0.378*	
C111	−0.0145 (13)	0.5127 (14)	0.3718 (8)	0.224 (7)	
H11D	−0.031274	0.468365	0.369814	0.269*	
H11E	−0.064575	0.552761	0.352880	0.269*	
C112	0.0790 (11)	0.5132 (13)	0.3412 (7)	0.212 (7)	
H12D	0.105726	0.548618	0.354657	0.255*	
H12E	0.123789	0.466463	0.350235	0.255*	
C113	0.0558 (11)	0.5319 (12)	0.2743 (7)	0.227 (7)	
H13G	0.003583	0.513112	0.265207	0.272*	
H13H	0.038084	0.583526	0.261809	0.272*	
C114	0.1423 (14)	0.4991 (10)	0.2421 (6)	0.228 (7)	
H14G	0.198173	0.498125	0.264986	0.273*	
H14H	0.143363	0.450384	0.237975	0.273*	
C115	0.1409 (14)	0.5431 (10)	0.1813 (8)	0.212 (7)	
H15G	0.101665	0.591265	0.182298	0.254*	
H15H	0.204700	0.545830	0.169075	0.254*	
C116	0.1020 (16)	0.5095 (10)	0.1376 (8)	0.184 (7)	
H16G	0.097601	0.538370	0.097422	0.276*	
H16H	0.039836	0.505215	0.151024	0.276*	
H16I	0.142876	0.462656	0.135533	0.276*	

### Atomic displacement parameters (Å^2^)

**Table:**

	*U*^11^	*U*^22^	*U*^33^	*U*^12^	*U*^13^	*U*^23^
Lu1	0.0200 (2)	0.0269 (2)	0.0177 (2)	−0.01156 (18)	0.00297 (17)	−0.00276 (17)
Lu2	0.0197 (2)	0.0240 (2)	0.0181 (2)	−0.00758 (17)	0.00353 (17)	−0.00374 (17)
P1	0.0249 (14)	0.0231 (13)	0.0270 (13)	−0.0044 (10)	0.0046 (10)	−0.0017 (10)
P2	0.0199 (13)	0.0325 (13)	0.0247 (13)	−0.0124 (10)	0.0036 (10)	−0.0075 (10)
P3	0.0367 (16)	0.0247 (13)	0.0287 (14)	−0.0088 (11)	0.0059 (12)	−0.0005 (11)
P4	0.0332 (16)	0.0568 (18)	0.0258 (14)	−0.0258 (14)	0.0023 (11)	−0.0107 (12)
P5	0.0248 (14)	0.0312 (14)	0.0236 (13)	−0.0119 (11)	0.0056 (10)	−0.0030 (10)
P6	0.0232 (13)	0.0288 (13)	0.0198 (12)	−0.0070 (10)	0.0050 (10)	−0.0022 (10)
O1	0.018 (3)	0.030 (3)	0.032 (4)	−0.002 (3)	0.007 (3)	−0.004 (3)
O2	0.032 (4)	0.024 (3)	0.027 (3)	−0.006 (3)	0.010 (3)	0.000 (3)
O3	0.023 (3)	0.027 (3)	0.031 (4)	−0.006 (3)	0.007 (3)	−0.005 (3)
O4	0.035 (4)	0.030 (4)	0.023 (3)	−0.006 (3)	0.000 (3)	−0.004 (3)
O5	0.022 (3)	0.037 (4)	0.029 (4)	−0.012 (3)	0.002 (3)	−0.010 (3)
O6	0.020 (3)	0.033 (3)	0.027 (3)	−0.011 (3)	0.008 (3)	−0.009 (3)
O7	0.029 (4)	0.027 (3)	0.030 (4)	−0.002 (3)	0.004 (3)	−0.007 (3)
O8	0.023 (3)	0.030 (3)	0.023 (3)	−0.007 (3)	−0.001 (3)	−0.001 (3)
O9	0.030 (4)	0.029 (3)	0.035 (4)	−0.012 (3)	0.009 (3)	−0.004 (3)
O10	0.024 (4)	0.036 (4)	0.031 (4)	−0.009 (3)	0.014 (3)	0.002 (3)
O11	0.041 (4)	0.032 (4)	0.034 (4)	−0.014 (3)	0.002 (3)	−0.004 (3)
O12	0.027 (4)	0.029 (4)	0.033 (4)	−0.008 (3)	0.010 (3)	−0.003 (3)
O13	0.033 (4)	0.059 (4)	0.021 (3)	−0.023 (3)	0.006 (3)	−0.007 (3)
O14	0.032 (4)	0.043 (4)	0.022 (3)	−0.023 (3)	0.003 (3)	−0.014 (3)
O15	0.027 (4)	0.094 (6)	0.031 (4)	−0.015 (4)	−0.003 (3)	−0.008 (4)
O16	0.054 (5)	0.063 (5)	0.031 (4)	−0.035 (4)	0.000 (3)	−0.018 (3)
O17	0.017 (3)	0.036 (3)	0.027 (3)	−0.014 (3)	0.009 (3)	−0.004 (3)
O18	0.033 (4)	0.039 (4)	0.019 (3)	−0.016 (3)	0.003 (3)	−0.006 (3)
O19	0.025 (4)	0.028 (3)	0.030 (3)	−0.001 (3)	0.008 (3)	0.003 (3)
O20	0.027 (4)	0.033 (3)	0.019 (3)	−0.012 (3)	0.012 (3)	0.000 (3)
O21	0.028 (4)	0.029 (3)	0.019 (3)	−0.005 (3)	0.007 (3)	−0.004 (3)
O22	0.024 (3)	0.038 (4)	0.015 (3)	−0.005 (3)	0.007 (3)	−0.005 (3)
O23	0.016 (3)	0.046 (4)	0.024 (3)	−0.010 (3)	0.002 (3)	−0.012 (3)
O24	0.021 (3)	0.038 (4)	0.023 (3)	−0.010 (3)	0.004 (3)	−0.006 (3)
C1	0.036 (6)	0.029 (5)	0.032 (5)	−0.002 (4)	0.001 (4)	−0.014 (4)
C2	0.049 (7)	0.042 (6)	0.045 (6)	−0.017 (5)	−0.006 (5)	−0.015 (5)
C3	0.028 (5)	0.027 (5)	0.020 (5)	−0.007 (4)	0.002 (4)	−0.009 (4)
C4	0.024 (5)	0.036 (6)	0.025 (5)	−0.007 (4)	0.001 (4)	−0.005 (4)
C5	0.037 (6)	0.029 (5)	0.031 (6)	0.001 (5)	0.000 (5)	0.000 (4)
C6	0.043 (7)	0.028 (5)	0.037 (6)	−0.007 (5)	0.015 (5)	0.004 (4)
C7	0.029 (6)	0.030 (5)	0.034 (6)	−0.008 (4)	0.015 (4)	0.000 (4)
C8	0.031 (6)	0.028 (5)	0.019 (5)	−0.003 (4)	0.002 (4)	0.003 (4)
C9	0.036 (6)	0.034 (5)	0.032 (6)	−0.015 (5)	0.007 (4)	0.002 (4)
C10	0.026 (6)	0.060 (7)	0.051 (7)	−0.014 (5)	0.009 (5)	0.011 (6)
C11	0.017 (5)	0.052 (7)	0.059 (7)	−0.004 (5)	0.000 (5)	0.003 (5)
C12	0.039 (7)	0.049 (7)	0.087 (9)	−0.016 (5)	0.004 (6)	−0.024 (6)
C13	0.053 (8)	0.039 (7)	0.077 (9)	−0.002 (6)	0.021 (6)	0.013 (6)
C14	0.027 (6)	0.032 (5)	0.034 (6)	−0.006 (4)	0.009 (4)	0.005 (4)
C15	0.028 (6)	0.038 (6)	0.025 (5)	−0.001 (4)	0.010 (4)	0.001 (4)
C16	0.037 (6)	0.032 (6)	0.045 (6)	−0.005 (5)	0.013 (5)	0.008 (5)
C17	0.033 (6)	0.039 (6)	0.034 (6)	−0.020 (5)	0.015 (5)	−0.006 (4)
C18	0.034 (7)	0.060 (7)	0.043 (7)	0.012 (5)	−0.007 (5)	−0.006 (6)
C19	0.069 (9)	0.048 (7)	0.070 (9)	−0.005 (6)	−0.030 (7)	0.008 (6)
C20	0.018 (5)	0.027 (5)	0.028 (5)	−0.011 (4)	0.001 (4)	−0.008 (4)
C21	0.035 (6)	0.024 (5)	0.021 (5)	−0.013 (4)	−0.002 (4)	−0.003 (4)
C22	0.044 (7)	0.053 (6)	0.026 (5)	−0.033 (5)	0.006 (5)	−0.015 (5)
C23	0.044 (7)	0.041 (6)	0.033 (6)	−0.023 (5)	−0.008 (5)	−0.004 (5)
C24	0.027 (6)	0.048 (6)	0.031 (6)	−0.017 (5)	0.007 (4)	−0.009 (5)
C25	0.036 (6)	0.039 (6)	0.015 (5)	−0.020 (5)	0.000 (4)	−0.007 (4)
C26	0.039 (6)	0.034 (5)	0.022 (5)	−0.013 (5)	0.005 (4)	−0.007 (4)
C27	0.029 (6)	0.039 (6)	0.034 (5)	−0.017 (4)	0.011 (4)	−0.007 (4)
C28	0.038 (6)	0.046 (6)	0.028 (5)	−0.010 (5)	0.003 (5)	−0.004 (5)
C29	0.043 (7)	0.054 (7)	0.031 (6)	−0.013 (5)	0.008 (5)	−0.010 (5)
C30	0.047 (7)	0.081 (8)	0.033 (6)	−0.040 (6)	−0.011 (5)	0.003 (6)
C31	0.029 (6)	0.051 (6)	0.031 (5)	−0.023 (5)	−0.001 (4)	−0.006 (5)
C32	0.059 (7)	0.058 (7)	0.035 (6)	−0.040 (6)	0.016 (5)	−0.002 (5)
C33	0.042 (7)	0.100 (9)	0.042 (7)	−0.042 (7)	0.017 (5)	−0.032 (6)
C34	0.058 (8)	0.073 (8)	0.028 (6)	−0.035 (6)	−0.001 (5)	0.018 (5)
C35	0.039 (7)	0.042 (6)	0.056 (7)	−0.011 (5)	0.004 (5)	−0.009 (5)
C36	0.113 (11)	0.094 (10)	0.143 (12)	−0.057 (9)	0.050 (10)	−0.056 (9)
C37	0.028 (5)	0.018 (5)	0.029 (5)	−0.005 (4)	0.004 (4)	−0.001 (4)
C38	0.017 (5)	0.029 (5)	0.034 (5)	−0.002 (4)	−0.008 (4)	−0.011 (4)
C39	0.033 (6)	0.033 (5)	0.030 (5)	−0.011 (4)	−0.001 (4)	−0.001 (4)
C40	0.034 (6)	0.037 (6)	0.035 (6)	−0.004 (5)	0.002 (5)	−0.005 (5)
C41	0.049 (7)	0.022 (5)	0.037 (6)	0.005 (5)	0.002 (5)	−0.016 (4)
C42	0.025 (5)	0.029 (5)	0.040 (6)	0.001 (4)	0.005 (4)	−0.007 (4)
C43	0.027 (5)	0.031 (5)	0.032 (5)	−0.011 (4)	0.013 (4)	−0.007 (4)
C44	0.031 (6)	0.032 (5)	0.035 (6)	−0.007 (4)	0.006 (4)	−0.010 (4)
C45	0.028 (6)	0.032 (5)	0.034 (5)	−0.009 (4)	0.009 (4)	−0.010 (4)
C46	0.052 (7)	0.036 (6)	0.024 (5)	−0.009 (5)	−0.001 (5)	−0.003 (4)
C47	0.056 (8)	0.037 (6)	0.070 (8)	−0.009 (6)	0.001 (6)	−0.012 (6)
C48	0.040 (6)	0.038 (6)	0.033 (6)	−0.002 (5)	0.007 (5)	−0.013 (5)
C49	0.044 (7)	0.055 (7)	0.052 (7)	0.005 (6)	0.005 (6)	−0.021 (6)
C50	0.058 (8)	0.096 (10)	0.035 (7)	−0.014 (7)	0.012 (6)	−0.012 (6)
C51	0.039 (7)	0.059 (7)	0.042 (6)	−0.003 (5)	0.026 (5)	0.000 (5)
C52	0.056 (8)	0.060 (8)	0.062 (8)	−0.005 (6)	−0.012 (7)	−0.008 (6)
C53	0.097 (11)	0.087 (10)	0.071 (9)	−0.022 (9)	−0.054 (8)	0.006 (8)
C54	0.040 (7)	0.053 (7)	0.034 (6)	−0.011 (5)	−0.003 (5)	−0.017 (5)
C55	0.020 (6)	0.050 (7)	0.054 (7)	−0.012 (5)	0.003 (5)	−0.015 (5)
C56	0.020 (5)	0.037 (6)	0.042 (6)	−0.006 (4)	−0.005 (5)	−0.002 (5)
C57	0.031 (6)	0.052 (7)	0.033 (6)	−0.026 (5)	−0.005 (5)	−0.009 (5)
C58	0.053 (8)	0.074 (8)	0.040 (7)	−0.033 (6)	0.003 (6)	−0.007 (6)
C59	0.044 (7)	0.059 (7)	0.041 (7)	−0.021 (6)	−0.003 (5)	−0.010 (5)
C60	0.025 (6)	0.047 (6)	0.038 (6)	−0.010 (5)	0.011 (4)	−0.019 (5)
C61	0.041 (7)	0.055 (7)	0.049 (7)	−0.022 (5)	0.002 (5)	−0.014 (5)
C62	0.036 (6)	0.055 (7)	0.038 (6)	−0.004 (5)	0.005 (5)	−0.007 (5)
C63	0.024 (6)	0.087 (9)	0.051 (7)	−0.018 (6)	0.017 (5)	−0.010 (6)
C64	0.056 (8)	0.068 (8)	0.044 (7)	−0.030 (6)	−0.018 (6)	−0.006 (6)
C65	0.025 (6)	0.065 (8)	0.061 (8)	−0.005 (5)	0.011 (5)	−0.025 (6)
C66	0.053 (8)	0.072 (8)	0.047 (7)	−0.034 (6)	0.022 (6)	−0.017 (6)
C67	0.036 (7)	0.063 (8)	0.064 (8)	0.005 (6)	0.009 (6)	0.005 (6)
C68	0.089 (11)	0.093 (11)	0.124 (13)	−0.026 (9)	0.016 (10)	−0.074 (10)
C69	0.037 (6)	0.034 (6)	0.040 (6)	−0.003 (5)	−0.003 (5)	0.001 (5)
C70	0.046 (7)	0.045 (7)	0.054 (7)	0.003 (5)	0.004 (6)	−0.009 (6)
C71	0.027 (5)	0.021 (5)	0.026 (5)	−0.007 (4)	0.012 (4)	0.000 (4)
C72	0.036 (6)	0.015 (5)	0.034 (6)	−0.001 (4)	0.009 (4)	0.006 (4)
C73	0.051 (7)	0.022 (5)	0.029 (5)	−0.002 (5)	0.013 (5)	0.002 (4)
C74	0.055 (7)	0.037 (6)	0.039 (7)	−0.015 (5)	0.020 (5)	−0.001 (5)
C75	0.026 (6)	0.028 (5)	0.055 (7)	−0.007 (4)	0.014 (5)	0.004 (5)
C76	0.032 (6)	0.029 (5)	0.036 (6)	−0.006 (4)	0.006 (5)	−0.001 (4)
C77	0.038 (6)	0.039 (6)	0.023 (5)	−0.018 (5)	0.009 (4)	0.003 (4)
C78	0.040 (6)	0.030 (5)	0.037 (6)	−0.015 (5)	−0.001 (5)	−0.004 (4)
C79	0.061 (8)	0.065 (7)	0.018 (5)	−0.022 (6)	0.004 (5)	−0.002 (5)
C80	0.046 (7)	0.032 (6)	0.037 (6)	−0.013 (5)	0.013 (5)	−0.006 (5)
C81	0.059 (8)	0.065 (8)	0.034 (6)	−0.017 (6)	0.032 (5)	−0.009 (5)
C82	0.024 (5)	0.046 (6)	0.038 (6)	−0.022 (5)	0.007 (4)	0.000 (5)
C83	0.033 (6)	0.052 (7)	0.037 (6)	−0.021 (5)	0.003 (5)	−0.009 (5)
C84	0.044 (7)	0.051 (7)	0.053 (7)	−0.032 (5)	0.010 (5)	0.004 (5)
C85	0.040 (6)	0.044 (6)	0.038 (6)	−0.020 (5)	0.004 (5)	−0.013 (5)
C86	0.029 (6)	0.080 (8)	0.024 (5)	−0.028 (5)	0.010 (4)	−0.019 (5)
C87	0.032 (6)	0.046 (6)	0.037 (6)	−0.007 (5)	0.000 (5)	−0.005 (5)
C88	0.026 (5)	0.037 (5)	0.015 (5)	−0.009 (4)	0.006 (4)	−0.009 (4)
C89	0.025 (5)	0.036 (5)	0.019 (5)	−0.010 (4)	0.003 (4)	−0.009 (4)
C90	0.032 (6)	0.029 (5)	0.025 (5)	−0.014 (4)	0.002 (4)	0.001 (4)
C91	0.028 (6)	0.068 (7)	0.021 (5)	−0.021 (5)	0.012 (4)	−0.012 (5)
C92	0.017 (5)	0.051 (6)	0.026 (5)	−0.006 (4)	−0.002 (4)	−0.011 (5)
C93	0.017 (5)	0.032 (5)	0.029 (5)	−0.005 (4)	0.005 (4)	−0.007 (4)
C94	0.035 (6)	0.029 (5)	0.034 (6)	−0.009 (4)	−0.004 (4)	−0.004 (4)
C95	0.035 (6)	0.046 (6)	0.035 (6)	−0.005 (5)	−0.008 (5)	−0.003 (5)
C96	0.062 (8)	0.038 (6)	0.035 (6)	−0.009 (5)	−0.010 (5)	0.002 (5)
C97	0.055 (7)	0.023 (5)	0.036 (6)	−0.006 (5)	0.010 (5)	0.000 (4)
C98	0.035 (6)	0.072 (8)	0.036 (6)	−0.015 (6)	0.015 (5)	−0.014 (6)
C99	0.044 (7)	0.039 (6)	0.033 (6)	−0.009 (5)	0.006 (5)	−0.011 (5)
C100	0.034 (6)	0.042 (6)	0.037 (6)	0.003 (5)	−0.005 (5)	0.006 (5)
C101	0.090 (10)	0.032 (6)	0.065 (8)	−0.005 (6)	0.016 (7)	−0.015 (6)
C102	0.053 (7)	0.054 (7)	0.035 (6)	−0.021 (6)	0.002 (5)	−0.008 (5)
C103	0.078 (11)	0.176 (17)	0.076 (11)	−0.041 (11)	0.011 (9)	−0.032 (11)
C104	0.129 (14)	0.077 (10)	0.057 (9)	−0.032 (10)	−0.005 (9)	−0.010 (8)
C105	0.088 (11)	0.074 (9)	0.064 (9)	−0.022 (8)	−0.018 (8)	−0.010 (8)
C106	0.047 (8)	0.058 (8)	0.072 (9)	−0.008 (6)	−0.013 (7)	−0.023 (7)
C107	0.060 (8)	0.058 (8)	0.052 (8)	−0.008 (6)	−0.011 (6)	−0.020 (6)
C108	0.075 (10)	0.093 (10)	0.042 (7)	−0.014 (8)	−0.012 (7)	−0.008 (7)
C109	0.072 (10)	0.087 (10)	0.072 (10)	−0.015 (8)	0.009 (8)	−0.011 (8)
C110	0.351 (19)	0.195 (13)	0.226 (17)	−0.100 (15)	−0.050 (17)	−0.022 (15)
C111	0.325 (17)	0.177 (11)	0.196 (15)	−0.105 (13)	−0.051 (15)	−0.026 (13)
C112	0.289 (16)	0.160 (10)	0.210 (14)	−0.089 (12)	−0.049 (14)	−0.026 (12)
C113	0.277 (16)	0.172 (11)	0.237 (15)	−0.079 (12)	−0.016 (14)	−0.008 (12)
C114	0.266 (15)	0.179 (12)	0.247 (15)	−0.091 (11)	−0.006 (13)	−0.006 (11)
C115	0.244 (15)	0.175 (12)	0.230 (15)	−0.100 (11)	0.018 (13)	−0.005 (11)
C116	0.231 (16)	0.154 (13)	0.207 (16)	−0.129 (11)	0.031 (14)	−0.022 (12)

### Geometric parameters (Å, º)

**Table:**

Lu1—O1	2.222 (5)	C51—H51B	0.9800
Lu1—O5	2.196 (5)	C51—H51C	0.9800
Lu1—O9	2.193 (6)	C52—C53	1.483 (15)
Lu1—O13	2.172 (6)	C52—H52A	0.9900
Lu1—O17	2.280 (5)	C52—H52B	0.9900
Lu1—O18	2.274 (6)	C53—H53A	0.9800
Lu2—O2	2.192 (5)	C53—H53B	0.9800
Lu2—O6	2.216 (6)	C53—H53C	0.9800
Lu2—O10	2.178 (5)	C54—C55	1.425 (13)
Lu2—O14	2.200 (6)	C54—C59	1.426 (13)
Lu2—O21	2.264 (5)	C55—C56	1.391 (13)
Lu2—O22	2.276 (5)	C55—C60	1.532 (13)
P1—O2	1.494 (6)	C56—C57	1.337 (12)
P1—O1	1.496 (6)	C56—H56	0.9500
P1—O3	1.554 (6)	C57—C58	1.375 (13)
P1—O4	1.589 (6)	C57—C64	1.492 (13)
P2—O5	1.493 (6)	C58—C59	1.408 (14)
P2—O6	1.496 (6)	C58—H58	0.9500
P2—O7	1.557 (6)	C59—C65	1.544 (14)
P2—O8	1.583 (6)	C60—C62	1.536 (12)
P3—O10	1.500 (6)	C60—C61	1.545 (13)
P3—O9	1.501 (6)	C60—C63	1.546 (12)
P3—O11	1.571 (6)	C61—H61A	0.9800
P3—O12	1.590 (6)	C61—H61B	0.9800
P4—O14	1.500 (6)	C61—H61C	0.9800
P4—O13	1.500 (6)	C62—H62A	0.9800
P4—O16	1.544 (7)	C62—H62B	0.9800
P4—O15	1.571 (7)	C62—H62C	0.9800
P5—O18	1.502 (6)	C63—H63A	0.9800
P5—O17	1.505 (6)	C63—H63B	0.9800
P5—O20	1.561 (6)	C63—H63C	0.9800
P5—O19	1.570 (6)	C64—H64A	0.9800
P6—O22	1.496 (6)	C64—H64B	0.9800
P6—O21	1.504 (6)	C64—H64C	0.9800
P6—O24	1.568 (6)	C65—C67	1.501 (14)
P6—O23	1.587 (6)	C65—C68	1.567 (15)
O3—C1	1.471 (9)	C65—C66	1.581 (14)
O4—C3	1.439 (10)	C66—H66A	0.9800
O7—C18	1.427 (10)	C66—H66B	0.9800
O8—C20	1.409 (10)	C66—H66C	0.9800
O11—C35	1.442 (11)	C67—H67A	0.9800
O12—C37	1.416 (9)	C67—H67B	0.9800
O15—C52	1.520 (12)	C67—H67C	0.9800
O16—C54	1.440 (11)	C68—H68A	0.9800
O19—C69	1.435 (10)	C68—H68B	0.9800
O20—C71	1.411 (9)	C68—H68C	0.9800
O23—C86	1.430 (10)	C69—C70	1.479 (13)
O24—C88	1.439 (9)	C69—H69A	0.9900
C1—C2	1.483 (12)	C69—H69B	0.9900
C1—H1A	0.9900	C70—H70A	0.9800
C1—H1B	0.9900	C70—H70B	0.9800
C2—H2A	0.9800	C70—H70C	0.9800
C2—H2B	0.9800	C71—C76	1.382 (12)
C2—H2C	0.9800	C71—C72	1.410 (12)
C3—C4	1.388 (11)	C72—C73	1.387 (12)
C3—C8	1.412 (11)	C72—C77	1.551 (13)
C4—C5	1.387 (12)	C73—C74	1.375 (14)
C4—C9	1.546 (12)	C73—H73	0.9500
C5—C6	1.380 (12)	C74—C75	1.380 (14)
C5—H5	0.9500	C74—C81	1.530 (12)
C6—C7	1.370 (12)	C75—C76	1.401 (12)
C6—C13	1.512 (12)	C75—H75	0.9500
C7—C8	1.391 (11)	C76—C82	1.555 (13)
C7—H7	0.9500	C77—C79	1.533 (12)
C8—C14	1.545 (12)	C77—C80	1.547 (12)
C9—C12	1.519 (13)	C77—C78	1.549 (12)
C9—C11	1.531 (12)	C78—H78A	0.9800
C9—C10	1.541 (13)	C78—H78B	0.9800
C10—H10A	0.9800	C78—H78C	0.9800
C10—H10B	0.9800	C79—H79A	0.9800
C10—H10C	0.9800	C79—H79B	0.9800
C11—H11A	0.9800	C79—H79C	0.9800
C11—H11B	0.9800	C80—H80A	0.9800
C11—H11C	0.9800	C80—H80B	0.9800
C12—H12A	0.9800	C80—H80C	0.9800
C12—H12B	0.9800	C81—H81A	0.9800
C12—H12C	0.9800	C81—H81B	0.9800
C13—H13A	0.9800	C81—H81C	0.9800
C13—H13B	0.9800	C82—C84	1.514 (12)
C13—H13C	0.9800	C82—C85	1.533 (12)
C14—C15	1.512 (12)	C82—C83	1.534 (12)
C14—C17	1.529 (12)	C83—H83A	0.9800
C14—C16	1.540 (11)	C83—H83B	0.9800
C15—H15A	0.9800	C83—H83C	0.9800
C15—H15B	0.9800	C84—H84A	0.9800
C15—H15C	0.9800	C84—H84B	0.9800
C16—H16A	0.9800	C84—H84C	0.9800
C16—H16B	0.9800	C85—H85A	0.9800
C16—H16C	0.9800	C85—H85B	0.9800
C17—H17A	0.9800	C85—H85C	0.9800
C17—H17B	0.9800	C86—C87	1.509 (12)
C17—H17C	0.9800	C86—H86A	0.9900
C18—C19	1.446 (14)	C86—H86B	0.9900
C18—H18A	0.9900	C87—H87A	0.9800
C18—H18B	0.9900	C87—H87B	0.9800
C19—H19A	0.9800	C87—H87C	0.9800
C19—H19B	0.9800	C88—C93	1.380 (11)
C19—H19C	0.9800	C88—C89	1.403 (11)
C20—C21	1.396 (11)	C89—C90	1.391 (11)
C20—C25	1.398 (11)	C89—C94	1.549 (12)
C21—C22	1.384 (12)	C90—C91	1.390 (12)
C21—C26	1.577 (12)	C90—H90	0.9500
C22—C23	1.366 (13)	C91—C92	1.342 (12)
C22—H22	0.9500	C91—C98	1.540 (12)
C23—C24	1.391 (12)	C92—C93	1.395 (11)
C23—C30	1.514 (13)	C92—H92	0.9500
C24—C25	1.396 (12)	C93—C99	1.553 (12)
C24—H24	0.9500	C94—C95	1.517 (12)
C25—C31	1.555 (12)	C94—C97	1.529 (12)
C26—C28	1.521 (12)	C94—C96	1.567 (12)
C26—C29	1.533 (12)	C95—H95A	0.9800
C26—C27	1.534 (11)	C95—H95B	0.9800
C27—H27A	0.9800	C95—H95C	0.9800
C27—H27B	0.9800	C96—H96A	0.9800
C27—H27C	0.9800	C96—H96B	0.9800
C28—H28A	0.9800	C96—H96C	0.9800
C28—H28B	0.9800	C97—H97A	0.9800
C28—H28C	0.9800	C97—H97B	0.9800
C29—H29A	0.9800	C97—H97C	0.9800
C29—H29B	0.9800	C98—H98A	0.9800
C29—H29C	0.9800	C98—H98B	0.9800
C30—H30A	0.9800	C98—H98C	0.9800
C30—H30B	0.9800	C99—C100	1.517 (13)
C30—H30C	0.9800	C99—C102	1.543 (13)
C31—C34	1.514 (13)	C99—C101	1.557 (12)
C31—C33	1.527 (13)	C100—H10D	0.9800
C31—C32	1.539 (12)	C100—H10E	0.9800
C32—H32A	0.9800	C100—H10F	0.9800
C32—H32B	0.9800	C101—H10G	0.9800
C32—H32C	0.9800	C101—H10H	0.9800
C33—H33A	0.9800	C101—H10I	0.9800
C33—H33B	0.9800	C102—H10J	0.9800
C33—H33C	0.9800	C102—H10K	0.9800
C34—H34A	0.9800	C102—H10L	0.9800
C34—H34B	0.9800	C103—C104	1.479 (8)
C34—H34C	0.9800	C103—H13D	0.9800
C35—C36	1.501 (7)	C103—H13E	0.9800
C35—H35A	0.9900	C103—H13F	0.9800
C35—H35B	0.9900	C104—C105	1.494 (8)
C36—H36A	0.9800	C104—H14D	0.9900
C36—H36B	0.9800	C104—H14E	0.9900
C36—H36C	0.9800	C105—C106	1.482 (8)
C37—C42	1.407 (11)	C105—H15D	0.9900
C37—C38	1.422 (12)	C105—H15E	0.9900
C38—C39	1.398 (11)	C106—C107	1.491 (8)
C38—C43	1.524 (11)	C106—H16D	0.9900
C39—C40	1.385 (12)	C106—H16E	0.9900
C39—H39	0.9500	C107—C108	1.488 (8)
C40—C41	1.380 (12)	C107—H17D	0.9900
C40—C47	1.495 (12)	C107—H17E	0.9900
C41—C42	1.385 (12)	C108—C109	1.487 (8)
C41—H41	0.9500	C108—H18D	0.9900
C42—C48	1.558 (12)	C108—H18E	0.9900
C43—C46	1.536 (12)	C109—H19D	0.9800
C43—C44	1.538 (11)	C109—H19E	0.9800
C43—C45	1.548 (12)	C109—H19F	0.9800
C44—H44A	0.9800	C110—C111	1.530 (7)
C44—H44B	0.9800	C110—H10M	0.9800
C44—H44C	0.9800	C110—H10N	0.9800
C45—H45A	0.9800	C110—H10O	0.9800
C45—H45B	0.9800	C111—C112	1.524 (7)
C45—H45C	0.9800	C111—H11D	0.9900
C46—H46A	0.9800	C111—H11E	0.9900
C46—H46B	0.9800	C112—C113	1.515 (7)
C46—H46C	0.9800	C112—H12D	0.9900
C47—H47A	0.9800	C112—H12E	0.9900
C47—H47B	0.9800	C113—C114	1.495 (7)
C47—H47C	0.9800	C113—H13G	0.9900
C48—C50	1.511 (14)	C113—H13H	0.9900
C48—C49	1.519 (12)	C114—C115	1.491 (7)
C48—C51	1.542 (13)	C114—H14G	0.9900
C49—H49A	0.9800	C114—H14H	0.9900
C49—H49B	0.9800	C115—C116	1.489 (7)
C49—H49C	0.9800	C115—H15G	0.9900
C50—H50A	0.9800	C115—H15H	0.9900
C50—H50B	0.9800	C116—H16G	0.9800
C50—H50C	0.9800	C116—H16H	0.9800
C51—H51A	0.9800	C116—H16I	0.9800
			
O13—Lu1—O9	84.0 (2)	C48—C51—H51C	109.5
O13—Lu1—O5	114.0 (2)	H51A—C51—H51C	109.5
O9—Lu1—O5	84.5 (2)	H51B—C51—H51C	109.5
O13—Lu1—O1	82.3 (2)	C53—C52—O15	108.3 (10)
O9—Lu1—O1	158.5 (2)	C53—C52—H52A	110.0
O5—Lu1—O1	86.0 (2)	O15—C52—H52A	110.0
O13—Lu1—O18	91.9 (2)	C53—C52—H52B	110.0
O9—Lu1—O18	91.9 (2)	O15—C52—H52B	110.0
O5—Lu1—O18	153.2 (2)	H52A—C52—H52B	108.4
O1—Lu1—O18	105.0 (2)	C52—C53—H53A	109.5
O13—Lu1—O17	154.3 (2)	C52—C53—H53B	109.5
O9—Lu1—O17	102.0 (2)	H53A—C53—H53B	109.5
O5—Lu1—O17	91.5 (2)	C52—C53—H53C	109.5
O1—Lu1—O17	97.5 (2)	H53A—C53—H53C	109.5
O18—Lu1—O17	63.25 (19)	H53B—C53—H53C	109.5
O10—Lu2—O2	116.1 (2)	C55—C54—C59	123.3 (10)
O10—Lu2—O14	85.0 (2)	C55—C54—O16	119.7 (8)
O2—Lu2—O14	84.6 (2)	C59—C54—O16	116.8 (9)
O10—Lu2—O6	87.3 (2)	C56—C55—C54	113.5 (9)
O2—Lu2—O6	81.7 (2)	C56—C55—C60	121.0 (8)
O14—Lu2—O6	159.44 (19)	C54—C55—C60	125.4 (9)
O10—Lu2—O21	149.6 (2)	C57—C56—C55	126.0 (9)
O2—Lu2—O21	93.77 (19)	C57—C56—H56	117.0
O14—Lu2—O21	92.6 (2)	C55—C56—H56	117.0
O6—Lu2—O21	103.4 (2)	C56—C57—C58	118.3 (9)
O10—Lu2—O22	87.3 (2)	C56—C57—C64	122.5 (9)
O2—Lu2—O22	156.3 (2)	C58—C57—C64	118.9 (10)
O14—Lu2—O22	102.5 (2)	C57—C58—C59	122.8 (10)
O6—Lu2—O22	96.2 (2)	C57—C58—H58	118.6
O21—Lu2—O22	63.56 (19)	C59—C58—H58	118.6
O2—P1—O1	114.5 (3)	C58—C59—C54	114.6 (10)
O2—P1—O3	108.2 (3)	C58—C59—C65	121.0 (10)
O1—P1—O3	110.3 (3)	C54—C59—C65	124.3 (9)
O2—P1—O4	107.0 (3)	C55—C60—C62	112.6 (8)
O1—P1—O4	110.0 (3)	C55—C60—C61	111.2 (8)
O3—P1—O4	106.4 (3)	C62—C60—C61	109.5 (8)
O5—P2—O6	113.3 (3)	C55—C60—C63	110.6 (8)
O5—P2—O7	110.5 (3)	C62—C60—C63	106.6 (8)
O6—P2—O7	110.6 (3)	C61—C60—C63	106.0 (8)
O5—P2—O8	107.0 (3)	C60—C61—H61A	109.5
O6—P2—O8	111.1 (3)	C60—C61—H61B	109.5
O7—P2—O8	103.8 (3)	H61A—C61—H61B	109.5
O10—P3—O9	114.6 (3)	C60—C61—H61C	109.5
O10—P3—O11	107.9 (3)	H61A—C61—H61C	109.5
O9—P3—O11	111.3 (4)	H61B—C61—H61C	109.5
O10—P3—O12	106.3 (3)	C60—C62—H62A	109.5
O9—P3—O12	109.4 (3)	C60—C62—H62B	109.5
O11—P3—O12	107.0 (3)	H62A—C62—H62B	109.5
O14—P4—O13	114.0 (4)	C60—C62—H62C	109.5
O14—P4—O16	110.2 (4)	H62A—C62—H62C	109.5
O13—P4—O16	106.1 (4)	H62B—C62—H62C	109.5
O14—P4—O15	111.6 (4)	C60—C63—H63A	109.5
O13—P4—O15	109.1 (4)	C60—C63—H63B	109.5
O16—P4—O15	105.4 (4)	H63A—C63—H63B	109.5
O18—P5—O17	105.1 (3)	C60—C63—H63C	109.5
O18—P5—O20	112.9 (3)	H63A—C63—H63C	109.5
O17—P5—O20	109.8 (3)	H63B—C63—H63C	109.5
O18—P5—O19	112.2 (3)	C57—C64—H64A	109.5
O17—P5—O19	114.1 (3)	C57—C64—H64B	109.5
O20—P5—O19	103.0 (3)	H64A—C64—H64B	109.5
O22—P6—O21	105.7 (3)	C57—C64—H64C	109.5
O22—P6—O24	111.1 (3)	H64A—C64—H64C	109.5
O21—P6—O24	111.5 (3)	H64B—C64—H64C	109.5
O22—P6—O23	113.7 (3)	C67—C65—C59	113.4 (9)
O21—P6—O23	112.9 (3)	C67—C65—C68	107.6 (10)
O24—P6—O23	102.2 (3)	C59—C65—C68	108.7 (9)
P1—O1—Lu1	123.5 (3)	C67—C65—C66	112.8 (9)
P1—O2—Lu2	164.0 (3)	C59—C65—C66	109.3 (9)
C1—O3—P1	123.2 (5)	C68—C65—C66	104.4 (9)
C3—O4—P1	127.5 (5)	C65—C66—H66A	109.5
P2—O5—Lu1	164.4 (4)	C65—C66—H66B	109.5
P2—O6—Lu2	124.3 (3)	H66A—C66—H66B	109.5
C18—O7—P2	126.7 (6)	C65—C66—H66C	109.5
C20—O8—P2	126.2 (5)	H66A—C66—H66C	109.5
P3—O9—Lu1	127.5 (3)	H66B—C66—H66C	109.5
P3—O10—Lu2	158.5 (4)	C65—C67—H67A	109.5
C35—O11—P3	121.6 (6)	C65—C67—H67B	109.5
C37—O12—P3	124.7 (6)	H67A—C67—H67B	109.5
P4—O13—Lu1	162.0 (4)	C65—C67—H67C	109.5
P4—O14—Lu2	125.9 (3)	H67A—C67—H67C	109.5
C52—O15—P4	126.3 (6)	H67B—C67—H67C	109.5
C54—O16—P4	125.5 (6)	C65—C68—H68A	109.5
P5—O17—Lu1	95.6 (3)	C65—C68—H68B	109.5
P5—O18—Lu1	96.0 (3)	H68A—C68—H68B	109.5
C69—O19—P5	121.0 (5)	C65—C68—H68C	109.5
C71—O20—P5	131.2 (5)	H68A—C68—H68C	109.5
P6—O21—Lu2	95.5 (3)	H68B—C68—H68C	109.5
P6—O22—Lu2	95.2 (3)	O19—C69—C70	108.6 (8)
C86—O23—P6	117.8 (5)	O19—C69—H69A	110.0
C88—O24—P6	127.9 (5)	C70—C69—H69A	110.0
O3—C1—C2	107.9 (7)	O19—C69—H69B	110.0
O3—C1—H1A	110.1	C70—C69—H69B	110.0
C2—C1—H1A	110.1	H69A—C69—H69B	108.4
O3—C1—H1B	110.1	C69—C70—H70A	109.5
C2—C1—H1B	110.1	C69—C70—H70B	109.5
H1A—C1—H1B	108.4	H70A—C70—H70B	109.5
C1—C2—H2A	109.5	C69—C70—H70C	109.5
C1—C2—H2B	109.5	H70A—C70—H70C	109.5
H2A—C2—H2B	109.5	H70B—C70—H70C	109.5
C1—C2—H2C	109.5	C76—C71—C72	122.3 (8)
H2A—C2—H2C	109.5	C76—C71—O20	119.4 (8)
H2B—C2—H2C	109.5	C72—C71—O20	118.0 (8)
C4—C3—C8	125.2 (8)	C73—C72—C71	117.0 (9)
C4—C3—O4	116.3 (7)	C73—C72—C77	118.4 (8)
C8—C3—O4	118.5 (7)	C71—C72—C77	124.6 (8)
C5—C4—C3	115.0 (8)	C74—C73—C72	122.4 (9)
C5—C4—C9	119.5 (8)	C74—C73—H73	118.8
C3—C4—C9	125.4 (8)	C72—C73—H73	118.8
C6—C5—C4	123.1 (9)	C73—C74—C75	118.4 (9)
C6—C5—H5	118.4	C73—C74—C81	120.6 (10)
C4—C5—H5	118.4	C75—C74—C81	120.9 (10)
C7—C6—C5	117.6 (8)	C74—C75—C76	122.4 (9)
C7—C6—C13	121.9 (9)	C74—C75—H75	118.8
C5—C6—C13	120.5 (9)	C76—C75—H75	118.8
C6—C7—C8	124.5 (8)	C71—C76—C75	116.7 (9)
C6—C7—H7	117.7	C71—C76—C82	125.9 (8)
C8—C7—H7	117.7	C75—C76—C82	117.3 (8)
C7—C8—C3	113.2 (8)	C79—C77—C80	106.4 (8)
C7—C8—C14	118.9 (8)	C79—C77—C78	106.6 (8)
C3—C8—C14	127.7 (8)	C80—C77—C78	110.5 (7)
C12—C9—C11	106.5 (8)	C79—C77—C72	110.9 (7)
C12—C9—C10	111.3 (8)	C80—C77—C72	110.3 (7)
C11—C9—C10	106.3 (8)	C78—C77—C72	111.9 (7)
C12—C9—C4	112.2 (8)	C77—C78—H78A	109.5
C11—C9—C4	110.7 (7)	C77—C78—H78B	109.5
C10—C9—C4	109.6 (8)	H78A—C78—H78B	109.5
C9—C10—H10A	109.5	C77—C78—H78C	109.5
C9—C10—H10B	109.5	H78A—C78—H78C	109.5
H10A—C10—H10B	109.5	H78B—C78—H78C	109.5
C9—C10—H10C	109.5	C77—C79—H79A	109.5
H10A—C10—H10C	109.5	C77—C79—H79B	109.5
H10B—C10—H10C	109.5	H79A—C79—H79B	109.5
C9—C11—H11A	109.5	C77—C79—H79C	109.5
C9—C11—H11B	109.5	H79A—C79—H79C	109.5
H11A—C11—H11B	109.5	H79B—C79—H79C	109.5
C9—C11—H11C	109.5	C77—C80—H80A	109.5
H11A—C11—H11C	109.5	C77—C80—H80B	109.5
H11B—C11—H11C	109.5	H80A—C80—H80B	109.5
C9—C12—H12A	109.5	C77—C80—H80C	109.5
C9—C12—H12B	109.5	H80A—C80—H80C	109.5
H12A—C12—H12B	109.5	H80B—C80—H80C	109.5
C9—C12—H12C	109.5	C74—C81—H81A	109.5
H12A—C12—H12C	109.5	C74—C81—H81B	109.5
H12B—C12—H12C	109.5	H81A—C81—H81B	109.5
C6—C13—H13A	109.5	C74—C81—H81C	109.5
C6—C13—H13B	109.5	H81A—C81—H81C	109.5
H13A—C13—H13B	109.5	H81B—C81—H81C	109.5
C6—C13—H13C	109.5	C84—C82—C85	106.0 (8)
H13A—C13—H13C	109.5	C84—C82—C83	107.4 (8)
H13B—C13—H13C	109.5	C85—C82—C83	110.4 (8)
C15—C14—C17	110.1 (7)	C84—C82—C76	111.6 (8)
C15—C14—C16	105.3 (8)	C85—C82—C76	110.6 (7)
C17—C14—C16	106.8 (7)	C83—C82—C76	110.6 (8)
C15—C14—C8	111.3 (7)	C82—C83—H83A	109.5
C17—C14—C8	112.9 (8)	C82—C83—H83B	109.5
C16—C14—C8	110.0 (7)	H83A—C83—H83B	109.5
C14—C15—H15A	109.5	C82—C83—H83C	109.5
C14—C15—H15B	109.5	H83A—C83—H83C	109.5
H15A—C15—H15B	109.5	H83B—C83—H83C	109.5
C14—C15—H15C	109.5	C82—C84—H84A	109.5
H15A—C15—H15C	109.5	C82—C84—H84B	109.5
H15B—C15—H15C	109.5	H84A—C84—H84B	109.5
C14—C16—H16A	109.5	C82—C84—H84C	109.5
C14—C16—H16B	109.5	H84A—C84—H84C	109.5
H16A—C16—H16B	109.5	H84B—C84—H84C	109.5
C14—C16—H16C	109.5	C82—C85—H85A	109.5
H16A—C16—H16C	109.5	C82—C85—H85B	109.5
H16B—C16—H16C	109.5	H85A—C85—H85B	109.5
C14—C17—H17A	109.5	C82—C85—H85C	109.5
C14—C17—H17B	109.5	H85A—C85—H85C	109.5
H17A—C17—H17B	109.5	H85B—C85—H85C	109.5
C14—C17—H17C	109.5	O23—C86—C87	107.4 (7)
H17A—C17—H17C	109.5	O23—C86—H86A	110.2
H17B—C17—H17C	109.5	C87—C86—H86A	110.2
O7—C18—C19	112.5 (9)	O23—C86—H86B	110.2
O7—C18—H18A	109.1	C87—C86—H86B	110.2
C19—C18—H18A	109.1	H86A—C86—H86B	108.5
O7—C18—H18B	109.1	C86—C87—H87A	109.5
C19—C18—H18B	109.1	C86—C87—H87B	109.5
H18A—C18—H18B	107.8	H87A—C87—H87B	109.5
C18—C19—H19A	109.5	C86—C87—H87C	109.5
C18—C19—H19B	109.5	H87A—C87—H87C	109.5
H19A—C19—H19B	109.5	H87B—C87—H87C	109.5
C18—C19—H19C	109.5	C93—C88—C89	124.5 (7)
H19A—C19—H19C	109.5	C93—C88—O24	117.9 (7)
H19B—C19—H19C	109.5	C89—C88—O24	117.1 (7)
C21—C20—C25	122.3 (8)	C90—C89—C88	114.9 (8)
C21—C20—O8	118.5 (7)	C90—C89—C94	119.4 (8)
C25—C20—O8	119.2 (7)	C88—C89—C94	125.8 (7)
C22—C21—C20	116.6 (8)	C91—C90—C89	121.7 (8)
C22—C21—C26	118.3 (8)	C91—C90—H90	119.1
C20—C21—C26	125.1 (8)	C89—C90—H90	119.1
C23—C22—C21	123.3 (9)	C92—C91—C90	119.5 (8)
C23—C22—H22	118.3	C92—C91—C98	120.7 (9)
C21—C22—H22	118.3	C90—C91—C98	119.8 (9)
C22—C23—C24	117.8 (9)	C91—C92—C93	123.0 (9)
C22—C23—C30	121.5 (9)	C91—C92—H92	118.5
C24—C23—C30	120.6 (9)	C93—C92—H92	118.5
C23—C24—C25	122.3 (9)	C88—C93—C92	115.2 (8)
C23—C24—H24	118.9	C88—C93—C99	125.6 (7)
C25—C24—H24	118.9	C92—C93—C99	119.2 (8)
C24—C25—C20	116.2 (8)	C95—C94—C97	111.2 (8)
C24—C25—C31	118.4 (8)	C95—C94—C89	112.2 (7)
C20—C25—C31	125.4 (8)	C97—C94—C89	110.6 (7)
C28—C26—C29	105.8 (7)	C95—C94—C96	106.4 (8)
C28—C26—C27	110.0 (8)	C97—C94—C96	106.7 (7)
C29—C26—C27	108.3 (7)	C89—C94—C96	109.6 (7)
C28—C26—C21	112.9 (7)	C94—C95—H95A	109.5
C29—C26—C21	109.9 (7)	C94—C95—H95B	109.5
C27—C26—C21	109.8 (7)	H95A—C95—H95B	109.5
C26—C27—H27A	109.5	C94—C95—H95C	109.5
C26—C27—H27B	109.5	H95A—C95—H95C	109.5
H27A—C27—H27B	109.5	H95B—C95—H95C	109.5
C26—C27—H27C	109.5	C94—C96—H96A	109.5
H27A—C27—H27C	109.5	C94—C96—H96B	109.5
H27B—C27—H27C	109.5	H96A—C96—H96B	109.5
C26—C28—H28A	109.5	C94—C96—H96C	109.5
C26—C28—H28B	109.5	H96A—C96—H96C	109.5
H28A—C28—H28B	109.5	H96B—C96—H96C	109.5
C26—C28—H28C	109.5	C94—C97—H97A	109.5
H28A—C28—H28C	109.5	C94—C97—H97B	109.5
H28B—C28—H28C	109.5	H97A—C97—H97B	109.5
C26—C29—H29A	109.5	C94—C97—H97C	109.5
C26—C29—H29B	109.5	H97A—C97—H97C	109.5
H29A—C29—H29B	109.5	H97B—C97—H97C	109.5
C26—C29—H29C	109.5	C91—C98—H98A	109.5
H29A—C29—H29C	109.5	C91—C98—H98B	109.5
H29B—C29—H29C	109.5	H98A—C98—H98B	109.5
C23—C30—H30A	109.5	C91—C98—H98C	109.5
C23—C30—H30B	109.5	H98A—C98—H98C	109.5
H30A—C30—H30B	109.5	H98B—C98—H98C	109.5
C23—C30—H30C	109.5	C100—C99—C102	110.9 (8)
H30A—C30—H30C	109.5	C100—C99—C93	112.3 (8)
H30B—C30—H30C	109.5	C102—C99—C93	111.8 (8)
C34—C31—C33	110.7 (8)	C100—C99—C101	105.8 (8)
C34—C31—C32	107.2 (8)	C102—C99—C101	104.7 (8)
C33—C31—C32	107.3 (8)	C93—C99—C101	110.9 (8)
C34—C31—C25	112.8 (8)	C99—C100—H10D	109.5
C33—C31—C25	110.3 (8)	C99—C100—H10E	109.5
C32—C31—C25	108.3 (7)	H10D—C100—H10E	109.5
C31—C32—H32A	109.5	C99—C100—H10F	109.5
C31—C32—H32B	109.5	H10D—C100—H10F	109.5
H32A—C32—H32B	109.5	H10E—C100—H10F	109.5
C31—C32—H32C	109.5	C99—C101—H10G	109.5
H32A—C32—H32C	109.5	C99—C101—H10H	109.5
H32B—C32—H32C	109.5	H10G—C101—H10H	109.5
C31—C33—H33A	109.5	C99—C101—H10I	109.5
C31—C33—H33B	109.5	H10G—C101—H10I	109.5
H33A—C33—H33B	109.5	H10H—C101—H10I	109.5
C31—C33—H33C	109.5	C99—C102—H10J	109.5
H33A—C33—H33C	109.5	C99—C102—H10K	109.5
H33B—C33—H33C	109.5	H10J—C102—H10K	109.5
C31—C34—H34A	109.5	C99—C102—H10L	109.5
C31—C34—H34B	109.5	H10J—C102—H10L	109.5
H34A—C34—H34B	109.5	H10K—C102—H10L	109.5
C31—C34—H34C	109.5	C104—C103—H13D	109.5
H34A—C34—H34C	109.5	C104—C103—H13E	109.5
H34B—C34—H34C	109.5	H13D—C103—H13E	109.5
O11—C35—C36	110.8 (9)	C104—C103—H13F	109.5
O11—C35—H35A	109.5	H13D—C103—H13F	109.5
C36—C35—H35A	109.5	H13E—C103—H13F	109.5
O11—C35—H35B	109.5	C103—C104—C105	115.6 (11)
C36—C35—H35B	109.5	C103—C104—H14D	108.4
H35A—C35—H35B	108.1	C105—C104—H14D	108.4
C35—C36—H36A	109.5	C103—C104—H14E	108.4
C35—C36—H36B	109.5	C105—C104—H14E	108.4
H36A—C36—H36B	109.5	H14D—C104—H14E	107.4
C35—C36—H36C	109.5	C106—C105—C104	117.1 (11)
H36A—C36—H36C	109.5	C106—C105—H15D	108.0
H36B—C36—H36C	109.5	C104—C105—H15D	108.0
C42—C37—O12	118.7 (7)	C106—C105—H15E	108.0
C42—C37—C38	123.4 (8)	C104—C105—H15E	108.0
O12—C37—C38	117.9 (7)	H15D—C105—H15E	107.3
C39—C38—C37	114.2 (8)	C105—C106—C107	118.2 (10)
C39—C38—C43	118.7 (8)	C105—C106—H16D	107.8
C37—C38—C43	127.1 (8)	C107—C106—H16D	107.8
C40—C39—C38	124.2 (9)	C105—C106—H16E	107.8
C40—C39—H39	117.9	C107—C106—H16E	107.8
C38—C39—H39	117.9	H16D—C106—H16E	107.1
C41—C40—C39	117.7 (9)	C108—C107—C106	117.8 (10)
C41—C40—C47	121.6 (9)	C108—C107—H17D	107.9
C39—C40—C47	120.6 (9)	C106—C107—H17D	107.9
C40—C41—C42	123.0 (8)	C108—C107—H17E	107.9
C40—C41—H41	118.5	C106—C107—H17E	107.9
C42—C41—H41	118.5	H17D—C107—H17E	107.2
C41—C42—C37	116.2 (8)	C109—C108—C107	116.7 (11)
C41—C42—C48	117.8 (8)	C109—C108—H18D	108.1
C37—C42—C48	126.0 (8)	C107—C108—H18D	108.1
C38—C43—C46	110.4 (7)	C109—C108—H18E	108.1
C38—C43—C44	114.8 (7)	C107—C108—H18E	108.1
C46—C43—C44	105.5 (8)	H18D—C108—H18E	107.3
C38—C43—C45	108.7 (7)	C108—C109—H19D	109.5
C46—C43—C45	107.1 (7)	C108—C109—H19E	109.5
C44—C43—C45	110.1 (7)	H19D—C109—H19E	109.5
C43—C44—H44A	109.5	C108—C109—H19F	109.5
C43—C44—H44B	109.5	H19D—C109—H19F	109.5
H44A—C44—H44B	109.5	H19E—C109—H19F	109.5
C43—C44—H44C	109.5	C111—C110—H10M	109.5
H44A—C44—H44C	109.5	C111—C110—H10N	109.5
H44B—C44—H44C	109.5	H10M—C110—H10N	109.5
C43—C45—H45A	109.5	C111—C110—H10O	109.5
C43—C45—H45B	109.5	H10M—C110—H10O	109.5
H45A—C45—H45B	109.5	H10N—C110—H10O	109.5
C43—C45—H45C	109.5	C112—C111—C110	103.3 (8)
H45A—C45—H45C	109.5	C112—C111—H11D	111.1
H45B—C45—H45C	109.5	C110—C111—H11D	111.1
C43—C46—H46A	109.5	C112—C111—H11E	111.1
C43—C46—H46B	109.5	C110—C111—H11E	111.1
H46A—C46—H46B	109.5	H11D—C111—H11E	109.1
C43—C46—H46C	109.5	C113—C112—C111	104.0 (8)
H46A—C46—H46C	109.5	C113—C112—H12D	111.0
H46B—C46—H46C	109.5	C111—C112—H12D	111.0
C40—C47—H47A	109.5	C113—C112—H12E	111.0
C40—C47—H47B	109.5	C111—C112—H12E	111.0
H47A—C47—H47B	109.5	H12D—C112—H12E	109.0
C40—C47—H47C	109.5	C114—C113—C112	105.8 (8)
H47A—C47—H47C	109.5	C114—C113—H13G	110.6
H47B—C47—H47C	109.5	C112—C113—H13G	110.6
C50—C48—C49	108.5 (8)	C114—C113—H13H	110.6
C50—C48—C51	109.7 (9)	C112—C113—H13H	110.6
C49—C48—C51	106.5 (8)	H13G—C113—H13H	108.7
C50—C48—C42	111.0 (8)	C115—C114—C113	106.8 (8)
C49—C48—C42	108.4 (8)	C115—C114—H14G	110.4
C51—C48—C42	112.5 (7)	C113—C114—H14G	110.4
C48—C49—H49A	109.5	C115—C114—H14H	110.4
C48—C49—H49B	109.5	C113—C114—H14H	110.4
H49A—C49—H49B	109.5	H14G—C114—H14H	108.6
C48—C49—H49C	109.5	C116—C115—C114	107.6 (8)
H49A—C49—H49C	109.5	C116—C115—H15G	110.2
H49B—C49—H49C	109.5	C114—C115—H15G	110.2
C48—C50—H50A	109.5	C116—C115—H15H	110.2
C48—C50—H50B	109.5	C114—C115—H15H	110.2
H50A—C50—H50B	109.5	H15G—C115—H15H	108.5
C48—C50—H50C	109.5	C115—C116—H16G	109.5
H50A—C50—H50C	109.5	C115—C116—H16H	109.5
H50B—C50—H50C	109.5	H16G—C116—H16H	109.5
C48—C51—H51A	109.5	C115—C116—H16I	109.5
C48—C51—H51B	109.5	H16G—C116—H16I	109.5
H51A—C51—H51B	109.5	H16H—C116—H16I	109.5
